# Covalent functionalization by using blue light activated radicals: on the reaction mechanisms of arylazo sulfone binding on graphene†

**DOI:** 10.1039/d4na00359d

**Published:** 2024-08-01

**Authors:** Alessandro Mameli, Alessandro Kovtun, Derek Jones, Vasiliki Benekou, Vincenzo Palermo, Marco Bandini, Manuela Melucci

**Affiliations:** a Dipartimento di Chimica “Giacomo Ciamician” Alma Mater Studiorum – Università di Bologna Via P. Gobetti, 85 40129 Bologna Italy; b Istituto per la Sintesi Organica e la Fotoreattività (ISOF), Consiglio Nazionale delle Ricerche (CNR) Via P. Gobetti, 101 40129 Bologna Italy alessandro.kovtun@isof.cnr.it; c Dipartimento di Scienze Fisiche, Informatiche e Matematiche (FIM), Università di Modena e Reggio Emilia (UNIMORE) Via G. Campi, 213/A 41125 Modena Italy

## Abstract

Covalent functionalization of graphene presents a pivotal strategy to enhance its surface properties and overcome inherent chemical inertness. While diazonium salts have been extensively utilized for this purpose, their limitations necessitate exploration of alternative approaches. Arylazo sulfones, such as diazonium salt derivatives serving as chromophores, offer a promising solution, enabling photochemical reactions under visible light. Here, we propose a novel method for rapid covalent photofunctionalization of chemical vapor deposition (CVD) graphene on copper substrates using arylazo sulfones. The generation of aryl radicals – chlorobenzene in this case – was achieved through blue light LED irradiation of 4-chlorophenylazo methyl sulfone solution in acetonitrile. Efficient surface covalent modification of graphene was verified by observing (i) the photogeneration of radicals with a decrease in the π–π* band absorbance and an increase in the n–π* of arylazosulfone solution by UV-Vis spectroscopy; (ii) an increase in C sp^3^ defects on graphene from the Raman D band, the Auger C KLL signal and graphene C 1s X-ray photoelectron spectroscopy (XPS); and (iii) the presence of the chlorobenzene XPS Cl 2p signal. The aryl radical generation was enhanced by the copper substrate during irradiation, with a possible double path reaction mechanism. This approach highlights the versatility of arylazo sulfones in covalently patterning graphene surfaces, thus unlocking opportunities by overcoming the current approach consisting of the deposition of resist materials with successive cycles of lithography and electrochemistry.

## Abstract 2

Covalent functionalization of graphene presents a pivotal strategy to enhance its surface properties and overcome inherent chemical inertness.

## Graphical abstract

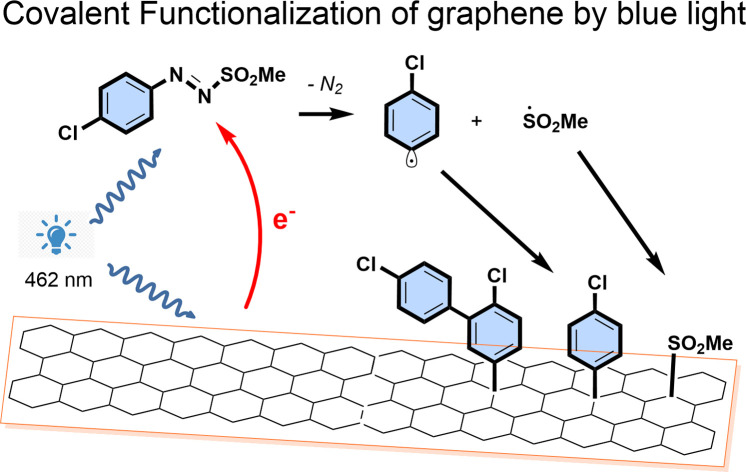

## Introduction

Due to its exceptional properties, graphene has been extensively investigated over the past two decades. Graphene is a promising candidate for applications in electronics^1,2^ and energy storage,^3,4^ thanks to its electrical conductivity. Moreover, its biocompatibility and large surface area make it appealing for biomedical uses, especially in drug delivery and biosensing.^5^ Graphene is commonly produced on a large scale using chemical vapor deposition (CVD) on copper and nickel substrates, which is advantageous for catalytic applications. Although this method provides control over parameters such as thickness, quality, and morphology, precise tuning of its properties through surface functionalization is needed to use it in a wider range of applications.

Surface functionalization of graphene and other 2D materials is now quite a well-established technique achieved through various methods, such as chemical activation,^6^ electrochemistry,^7,8^ thermal treatments,^9^ ultrasonication^10,11^ and more recently, visible-light photochemistry.^12^ Surface functionalization appears to be an even harder challenge considering the control of surface properties over limited spaces at the microscale, *i.e.* for the fabrication of multiarray sensors; only a few examples are present in the literature which involve the use of lithography and diazonium salts.

Diazonium salts are amongst the most versatile and widely used chemical vectors for the covalent functionalization of graphene and various other materials, including carbon compounds, metals, semiconductors, and polymers.^13^ This approach is based on a charge transfer mechanism, leading to the generation of aryl radicals (Ar˙) and their successive covalent bonding to the C sp^2^ lattice, followed by a change in graphitic carbon atom hybridization from sp^2^ to sp^3^.^14^ Among many, the most common approach relies on the use of an electrolytic cell, with graphene acting as a working electrode.^15^

In this framework, light-mediated covalent functionalization of 2D materials has received much attention over the past 15 years. One interesting approach is using visible-light laser (514 nm) photolysis of silver trifluoroacetate, generating a radical able to covalently bond to graphene.^16^ Although this approach seems to be effective and potentially scalable, metallic silver residues could affect the performance of any final device or sensor. In a previous study, similar irradiation (514.5 nm laser) successfully induced the covalent bonding of aryl groups onto graphene in only a few minutes using benzoyl peroxide in toluene. However, detailed mechanistic investigations revealed the pivotal light absorption by graphene with subsequent charge transfer to benzoyl peroxide.^17^ In fact, the direct photolysis of benzoyl was excluded since the compound does not adsorb in the visible region. More recently, the use of multiple wavelengths (532 nm, 450 nm and 610 nm) to produce intermediates for covalent bonding to graphene was also found to be suitable for the “classic” diazonium salts,^18^ where light-induced charge transfer (CT) was exploited for patterning on the microscale. However, uncontrolled covalent bonding occurred in non-irradiated areas; control experiments in the absence of light or with lower energy red light (610 nm) showed covalent bonding even without exposure to 532 nm green light or 450 nm blue light. Moreover, graphene uniformly absorbs ∼2.3% of light over the whole visible spectrum,^19^ and the photo-excited electrons with long wavelengths still have enough energy to overcome the reaction barrier.

In fact, the use of diazonium salts exhibits significant limitations for photo-induced covalent functionalization. As a matter of fact, even when functional groups containing oxygen or nitrogen are attached to the aromatic ring, these salts remain optically transparent to visible light. However, with sulfur-containing functional groups, in this case using 2,5-diethoxy-4-*n*-butylthiobenzenediazonium tetrafluoroborate, the diazonium salt shows a distinct absorption band around 400 nm.^20^ While this molecular modification offers an optical absorption advantage, it can also pose difficulties when a sulfur group is not desired in conjunction with the aryl cation. To address this issue, a sulfur-substituted diazo moiety –N_2_SO_2_CH_3_ has been proposed, as the N–S bond can be cleaved releasing nascent nitrogen. These compounds, known as arylazo sulfones, are chromophores which share a chemical structure similar to that of diazonium salts but exist in a covalent uncharged form. Originally studied in the early 1970s,^21^ their reaction mechanism was elucidated only more recently.^22,23^ Under the influence of heat or irradiation, arylazo sulfones can undergo decomposition or polymerization *via* either ionic or radical mechanisms, with the choice of mechanism favored in protic solvents (water) for the former and aprotic solvents (acetonitrile) for the latter.

These compounds have also found extensive use in metal-free coupling reactions both as radicals and electrophiles.^24–26^ More recently, they have been employed for photo-induced covalent bonding onto gold substrates,^27^ graphene oxide, and reduced graphene oxide surfaces.^12^ Similar photo-reactivity was also observed on aryldiazonium salts and arylazo sulfonates activated using blue light.^28^ Arylazo sulfones are not unique for photografting on surfaces, and also iodonium salts present analogous properties.^29^ Additionally, successful covalent bonding onto graphene was recently achieved through the solvation of diazonium cations with dimethyl sulfoxide (DMSO) anions, which share a molecular structure similar to that of the arylazo sulfones presented here.^30^

In fact, the present work introduces a quick method for the functionalization of CVD grown graphene on copper and graphite (Highly Oriented Pyrolytic Graphite, HOPG) surfaces with arylazo sulfones. The functionalization was investigated structurally and morphologically, with an emphasis on X-Ray Photoelectron Spectroscopy (XPS)^31^ and Raman spectroscopy.^32^ The influence of blue light and the presence of copper during the reactions were also investigated.

## Experimental

The 4-chlorophenylazo methyl sulfone (IUPAC name 1-(4-chlorophenyl)-2-(methylsulfonyl)diazene) used in the present work was synthesized from diazonium salts using the procedure described by Protti *et al.*^22^ as described in our previous work.^12^ Graphene monolayer films (CVD-G/Cu) on Cu were used as received (Graphenea, Spain). Highly Oriented Pyrolytic Graphite (HOPG) of ZYH grade (Advanced Ceramics, Cleveland, USA) was freshly cleaved before use.

The covalent functionalization of graphene and HOPG was achieved by irradiating the substrates in a 0.15 mM solution of 4-chlorophenylazo methyl sulfone in anhydrous acetonitrile with a commercial LED (462 nm) for 7 minutes under magnetic stirring. The temperature of the bath was kept below 30 °C and the irradiation power density of the photoreactor was 10–13 mW cm^−2^ as measured using a photoradiometer.

The control samples were prepared using the same reaction conditions but avoiding any exposure to either the LED or ambient light. From a previous study of diazonium salts on gold^33^ it might be expected that some reaction could occur spontaneously.

UV-Vis spectra of pre-irradiation and post-irradiation solutions of arylazo sulfones were recorded using a Cary 100 UV-Vis spectrophotometer (Agilent Technologies). The stability of the 4-chlorophenylazo methyl sulfone stock solution was checked daily and it showed stability for at least 15 days.

### Raman spectroscopy

Micro-Raman spectra of substrates were recorded using a LabRAM HR Evolution spectrometer (HORIBA Jobin Yvon Ltd), equipped with a grating (600 grooves per mm), combined with an Olympus BXFM-ILHS microscope. The samples were exposed to a source of 532 nm wavelength (Nd-YAG laser, nominal power 100 mW), with a maximum measured radiation power on the sample of less than 8 mW and a spatial resolution of <2 μm and analyzed with a 100× objective lens, resulting in a 0.5 μm sample diameter exposed to <0.8 mW incident power on the sample. The measurements were performed over a 100–3000 cm^−1^ spectral range with an overall acquisition time of 1.5 min distributed over five accumulations. The Raman spectrometer was calibrated using a silicon standard. For defect distance and density estimation on functionalized samples we have followed the criteria described in the literature.^34^

### X-ray photoelectron spectroscopy

XPS spectra were acquired using Al Kα radiation (ℏ*ω* = 1486.6 eV, 125 W) with a Phoibos 100 hemispherical electron energy analyzer (Specs). Spectra were recorded in the constant analyzer energy (CAE) mode with analyzer pass energies of 10 eV for the high-resolution spectra. An overall resolution of 0.9 eV was determined on freshly Ar^+^-sputtered silver measured on the Ag 3d_5/2_ peak. Base pressure in the analysis chamber during measurements was 3 × 10^−8^ mbar. More details on C 1s peak fitting are given in the ESI.† Auger signals were resampled in 0.2 eV steps and smoothed using ∼2 eV steps^35^ before numerical differentiation.

## Results and discussion

**Scheme 1:**
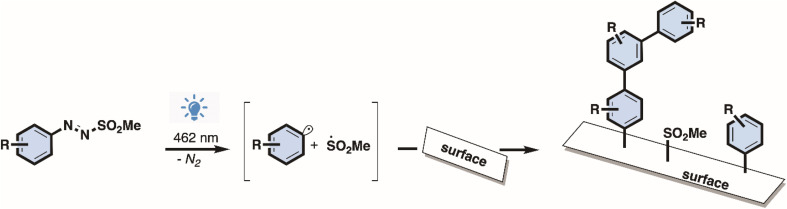
Arylazo sulfone surface functionalization mechanism.

Photo-induced covalent bonding of chlorobenzene onto graphene was achieved using a commercial LED, with an emission peak at 462 nm, *via* the generation of radicals from 4-chlorophenylazo methyl sulfone (Scheme 1).

Radical generation was monitored using UV-Vis spectroscopy, where the presence of π–π* transitions at 305 nm and n–π* at 425 nm is associated with the pristine molecule (Fig. SI 2†). The latter state is populated selectively with visible light exposure (462 nm here) with consequent N–S bond homolysis which, after nitrogen loss from the diazenyl radical 
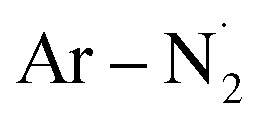
, generates an aryl (Ar˙)/methanesulfonyl 
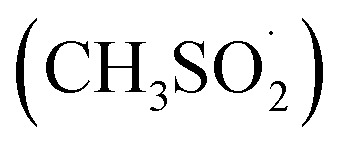
 radical pair.^23^ From the UV-Vis spectra it is possible to observe rapid degradation of the arylazo sulfone molecule over 5–10 minutes of LED irradiation, with a drastic decrease in the π–π* band and a flattening of the n–π* band. This implies that radical generation is relatively fast, with the reaction completing in only a few minutes. The kinetics observed by UV-Vis spectroscopy agree with previous results obtained on arylazo sulfonates irradiated at 456 nm,^28^ where the generation of aryl radicals was confirmed by Electron Paramagnetic Resonance (EPR) and TEMPO radical traps.

**Fig. 1:**
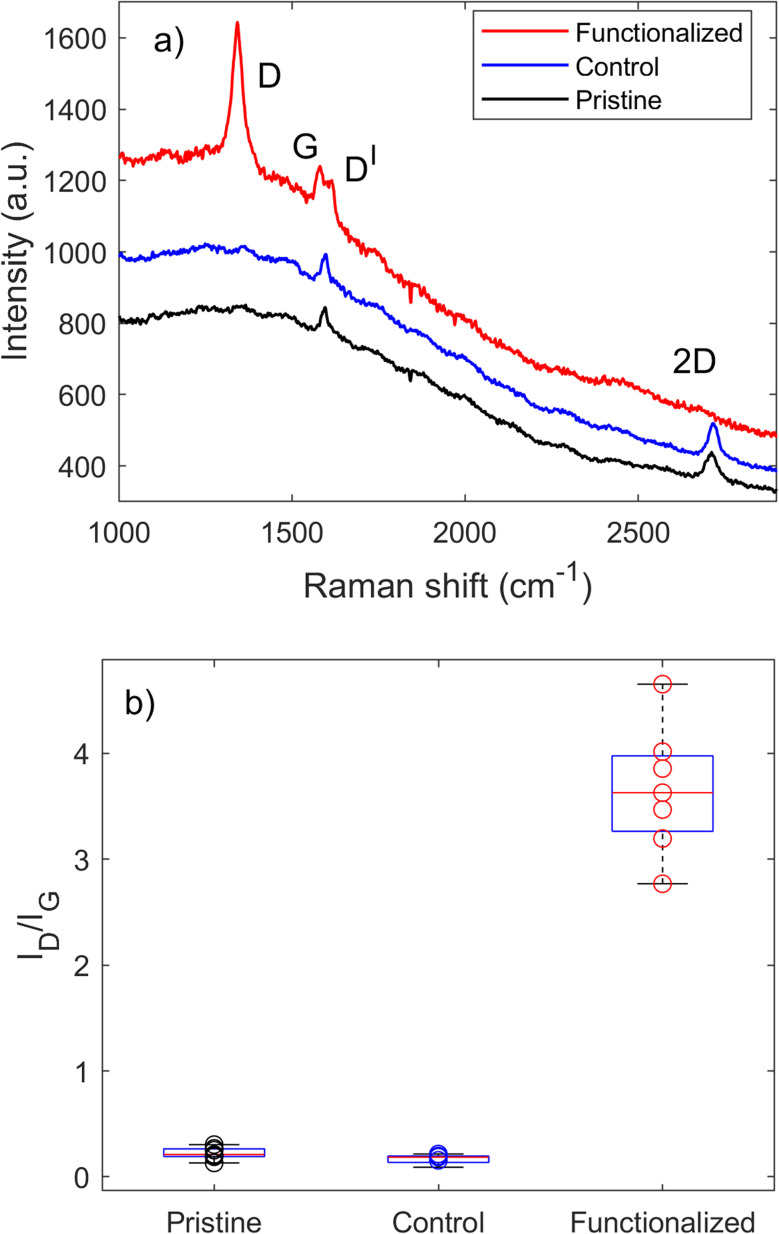
(a) Representative Raman spectra of pristine, control and functionalized CVD-G/Cu. (b) Box plot of *I*_D_/*I*_G_ of pristine, control and functionalized CVD-G/Cu. Functionalized samples were irradiated for 7 minutes in 0.15 mM solution of 4-chlorophenylazo methyl sulfone in acetonitrile.

The functionalization of CVD-G/Cu was monitored by Raman and XPS. The results from Raman spectroscopy are reported in Fig. 1. The Raman spectra of pristine CVD-G/Cu present the G (∼1595 cm^−1^) and 2D (∼2713 cm^−1^) bands, which depend on the laser frequency^36^ and correspond to the expected values for a 532 nm laser (2.33 eV). The typical D (∼1350 cm^−1^) peak associated with C sp^3^ defects on graphene gave an *I*_D_/*I*_G_ ratio of only 0.2, where *I*_D_ and *I*_G_ are the intensities of D and G peaks, respectively. The presence of single layer graphene was confirmed by observing the symmetry of the 2D peak, an FWHM of 38 cm^−1^ and the ratio between the areas of 2D and G peaks (*A*_2D_/*A*_G_ ratio) of ∼2.^37^

The covalent bonding of chlorobenzene onto CVD-G/Cu was confirmed by the appearance of an intense D peak, associated with the formation of C sp^3^ defects on graphene.^14^ The kinetics of the reaction was monitored by observing the evolution of the *I*_D_/*I*_G_ peak ratio. As reported in Fig. S11,† the behavior of *I*_D_/*I*_G_ as a function of time was in agreement with that expected for increasing defect density (or decreasing distance between defects).^34^ All the spectroscopic (XPS and Auger) and microscopic (AFM) characterization was performed on the sample with an *I*_D_/*I*_G_ peak ratio of 4, which was obtained after 7 minutes of reaction at a 0.15 mM concentration (see Table 1), with a high degree of functionalization being achieved within a relatively short time; longer reaction times lead to a further increase in functionalization, but after 15 minutes we observed a substantial plateau. It was possible to estimate that defect coverage after 7 minutes was of the order of magnitude of 1% and the corresponding distance between defects was close to 2 nm, similar to values found on chemical functionalization of different graphene-based materials.^6^ The control experiment was performed under the same reaction conditions, but without LED irradiation, presenting neither a D signal increase nor any other significant change in the Raman spectra. The initial *I*_D_/*I*_G_ value for pristine graphene of ∼0.2 was an overestimation, given the fluorescence signal present in the Raman signal. After functionalization, the D′ peak appeared at ∼1616 cm^−1^ with the 2D peak almost disappearing, in agreement with what is usually observed for highly functionalized graphene. The Raman spectrum of the dry powder of 4-chlorophenylazo methyl sulfone is reported in the ESI† with a tentative assignment to each vibrational Raman mode with no signal being observed in the D region (Fig. 1).

**Table 1:** Values of the main parameters obtained from Raman spectra of pristine, control and functionalized CVD-G/Cu

Parameter	Pristine	Control	Functionalized
D (cm^−1^)	1360 ± 4	1367 ± 7	1352 ± 7
G (cm^−1^)	1595 ± 8	1600 ± 4	1591 ± 5
2D (cm^−1^)	2713 ± 6	2712 ± 10	—
*I*_D_/*I*_G_	0.2 ± 0.1	0.19 ± 0.04	3.7 ± 0.6
FWHM 2D (cm^−1^)	38 ± 6	37 ± 5	—
*A*_2D_/*A*_G_	2.2 ± 0.4	1.7 ± 0.4	—

**Fig. 2:**
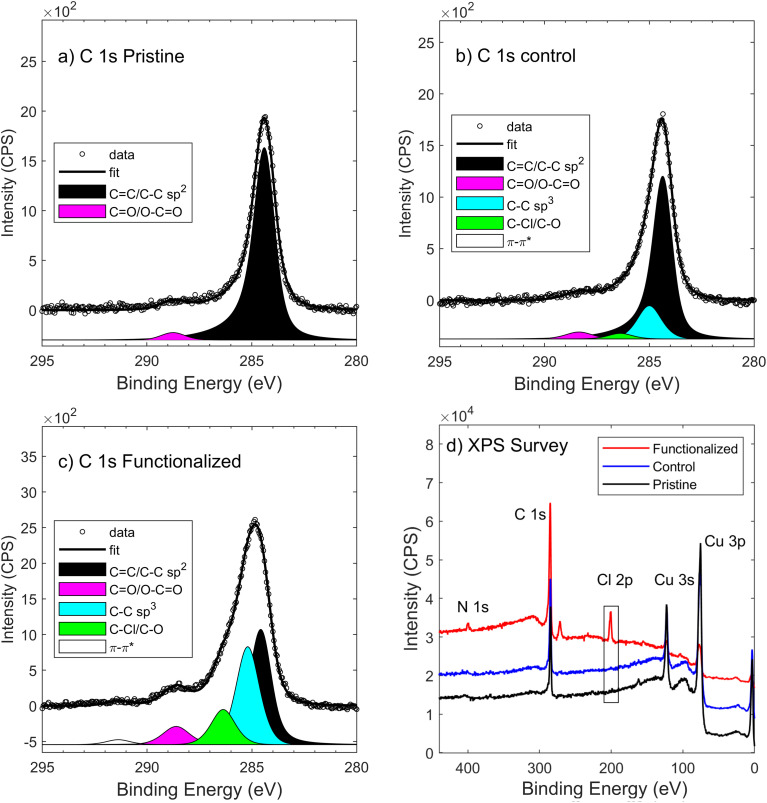
(a) XPS C 1s of pristine CVD-G/Cu. (b) XPS C 1s of control CVD-G/Cu. (c) XPS C 1s of functionalized CVD-G/Cu. (d) XPS survey spectra of pristine, control and functionalized graphene. The reduced chi square values of C 1s fit were: 4.0 (pristine), 2.9 (control) and 3.8 (functionalized).

**Fig. 3:**
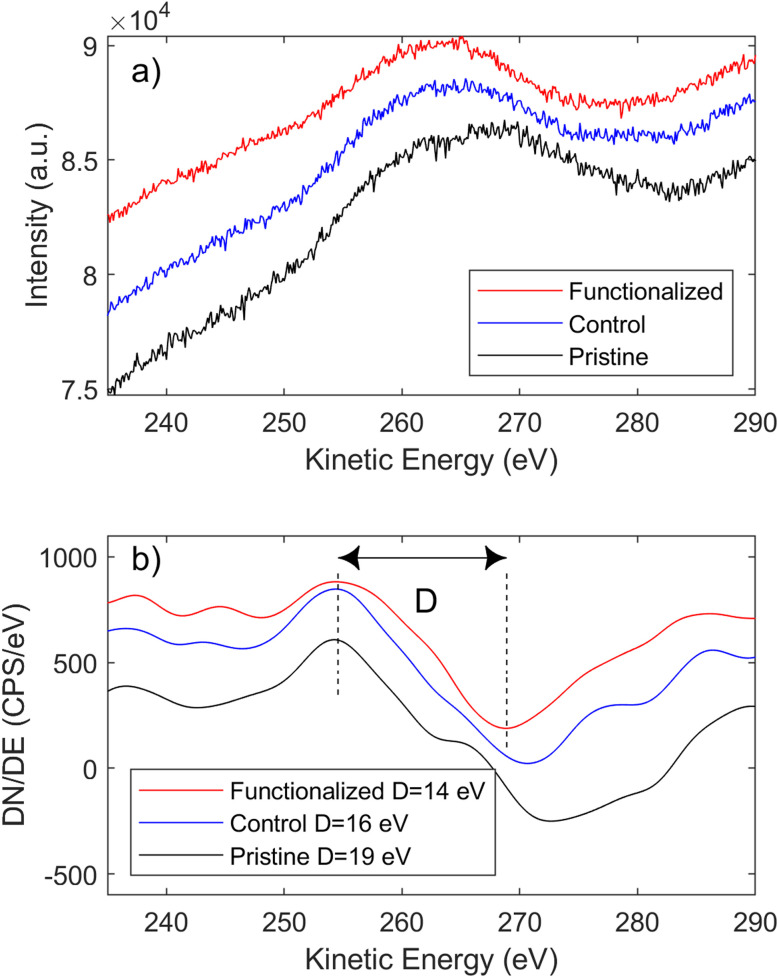
(a) C KLL Auger signal. (b) First-derivative of C KLL of pristine, control and functionalized graphene on copper.

The overall increase in C sp^3^ defects was also confirmed using XPS spectroscopy (Fig. 2), from the C 1s signals of graphene,^38^ as well as the degree of functionalization (Cl 2p signal from bonded chlorobenzene) and the attenuated signals from the Cu substrate (XPS is surface sensitive due to the limited photoelectron escape depth). The presence of C sp^3^ relative shift was confirmed by C 1s analysis for covalent functionalization on graphene;^39^ nevertheless, given the narrow chemical shift from the main component (C sp^2^), the quantification of C sp^3^ defects from C 1s fit is quite challenging and proper support of Auger signal analysis is necessary.^40^ On fitting the C 1s signal, the relative amount of C sp^2^ over all carbon atoms decreased from 97% to 46%, while the C KLL Auger signal (Fig. 3) confirmed the overall trend: the first-derivative of the C KLL signal shows a distance (in eV) between the relative minimum and maximum which decreases from 19 eV in pristine CVD-G/Cu to 14 eV in functionalized graphene, which is quite close to the 13.2 eV value reported for C sp^3^.^41^ This parameter is called D (delta), (not to be confused with the D band in Raman), which increases linearly with the amount of C sp^2^ present in the sample, as in the case of carbon nanotubes, amorphous diamonds and graphitic materials,^42^ although the presence of oxygen in C–O bonds may significantly affect the final C sp^2^ estimation using the D parameter, with some deviation from linearity, as observed by Lesiak.^41^ Moreover, the different kinetic energies of C KLL and C 1s electrons lead to different escape depths, with the C KLL electrons with ∼260 eV kinetic energy being more surface sensitive compared to the ∼1200 eV kinetic energy of the C 1s photoelectrons.^43,44^ Thus, both C KLL and C 1s signals confirmed the overall increase in C sp^3^, with the C KLL signal being much more sensitive to the C sp^3^ present in the upper layer formed by the grafted molecular layer (comprising aryl and methanesulfonyl groups^27^) compared to the C sp^3^ present on graphene itself, whereas the Raman signal derives only from C sp^3^ present on graphene.

XPS survey spectra (Fig. 2) also show strong Cl 2p_3/2_ signals peaking at 200.5 eV binding energy which arise from the chlorobenzene group (expected at 200.1 eV (ref. 45)) on functionalized samples, which are absent in control samples. The presence of chlorobenzene, combined with the results from the Raman spectroscopy (D band), confirms the effective covalent bonding of chlorobenzene onto graphene.

The XPS signals from the control samples seem to be affected by the reaction environment, contrary to what was observed in Raman data: C sp^3^ defects increase, as confirmed by both C KLL and C 1s, as well as by the O 1s signal increase (Table 2). The presence of the N 1s signal at ∼400 eV with 0.4 at% (Fig. S14†) could be associated with adsorbed arylazo sulfone degraded on the graphene surface during or after the control experiment (no Cl 2p or S 2p XPS signals were present), while it can be excluded in the presence of residues from acetonitrile trapped between graphene and copper (absence of the N 1s signal after 7 minutes of immersion of CVD-G/Cu in acetonitrile, and the spectrum was not reported). On the functionalized graphene samples the presence of significant amounts of nitrogen (2.0 at%) and chlorine (7.0 at%), from N 1s (∼400 eV) and Cl 2p signals, confirmed the presence of aryl (chlorobenzene) and nitrogen on the surface. The chemical state of nitrogen remains unclear: the broad signal (FWHM 3 eV) at *c.a.* 400 eV cannot be univocally associated with pyridinic (398.3 ± 0.3 eV), pyrrolic (400.1 ± 0.3 eV) or graphitic (401.5 ± 0.3 eV) nitrogen.^46^ We cannot exclude the covalent grafting of diazenyl radical 
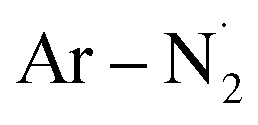
 to graphene with the –N=N– group as reported by Nicchio for arylazo sulfonate sodium salts (Ar–N=N–SO_3_Na^28^), but this reaction path would be secondary compared to the main one: given the relative amount of chlorine and nitrogen, we can estimate that only one aryl every seven is bonded to graphene *via* nitrogen (Ar–N=N–graphene), and the other six are directly bonded to the graphene (Ar–graphene). The sulfur content (S 2p_3/2_ ∼168 eV) on the surface is relatively low, ranging from 0.3 at% to 0.5 at%, indicating the presence of the methanesulfonyl group, due to the functionalization of graphene by the methanesulfonyl 
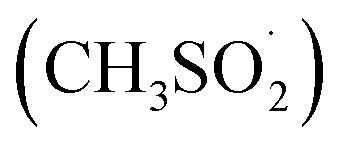
 radical. A related experiment, the covalent functionalization of gold substrates^27^ using arylazo sulfones, showed a comparable level of oxidized sulfur content and the presence of N–Au bonds. These considerations about the presence of heteroatoms (N, Cl and S) in functionalized graphene are in agreement with the main reaction path reported in the literature.^23^

**Table 2:** XPS binding energies (B. E. in eV), the associated chemical states and relative atomic composition (at%) of pristine, control and functionalized CVD-G/Cu

XPS signal	Binding energy (eV)	Chemical state	Pristine (at%)	Control (at%)	Functionalized (at%)
C 1s	284.4	C=C/C–C sp^2^	59.3	35.5	33.2
	285.1	C–C sp^3^	—	7.3	24.5
	286.3	C–Cl/C–O	—	1.4	9.3
	288.7	C=O/O–C=O	2.0	1.4	5.0
O 1s	532.0	O–C/Cu–OH	3.0	8.5	10.2
	530.6	O–Cu/O=C	1.7	17.2	2.4
Cu 2p_3/2_	932.8	Cu(0)	32.2	—	—
	932.3	Cu(i)	—	28.3	2.6
	935.0	Cu(ii)	—	0	3.3
Cl 2p_3/2_	200.5	Cl–C	—	—	7.0
N 1s	∼400	N–C	—	0.4	2.0
S 2p_3/2_	161.6	S–Cu	1.8	—	—
	168.6	S–O	—	—	0.5

XPS survey spectra also provide qualitative information on the increase in thickness of the carbon film (C 1s) on the copper substrate (Cu 2p): in pristine CVD-G/Cu the C/Cu ratio is close to 2, while after functionalization it increases to ∼12, due to a Cu 2p signal decrease and a C 1s increase. The average thickness of the carbon film – graphene and functionalization – on copper was obtained by using the XPS C/Cu ratio as proposed by Cumpson,^47^ obtaining a value in the range of 3.5 to 4 nm, at least 3 times larger than the initial thickness (pristine and control samples) of 0.8–1.5 nm. The obtained thickness is of the same order of magnitude as the previously reported result of arylazo sulfone on gold.^27^ These are average values calculated assuming a homogeneous film and considering only the major atoms present in the film (C) and substrate (Cu), thus excluding O, N and Cl. All these XPS results are compatible with a complete coverage of chlorobenzenes on graphene, where for each C–C sp^3^ defect on graphene (about 1% from the Raman results) there is a large number of chlorobenzenes attached in series.

**Fig. 4:**
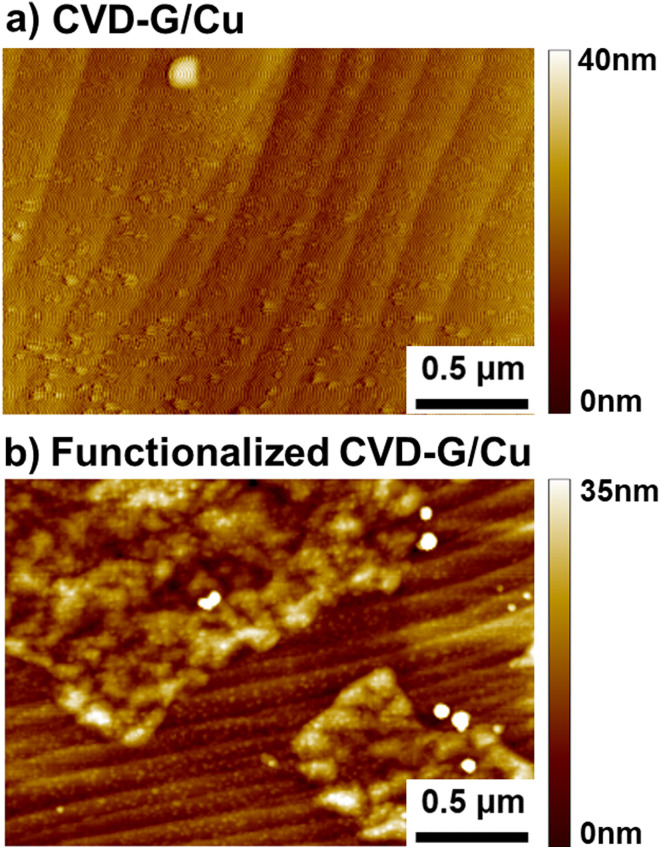
AFM topography of (a) pristine CVD-G/Cu and (b) functionalized CVD-G/Cu. The functionalized CVD-G/Cu presents both metallic Cu (horizontal lines) and oxidized regions.

The topography of both the pristine and the functionalized CVD-G/Cu was obtained *via* atomic force microscopy (Fig. 4). Both samples under study showed the typical terrace structures of the metallic copper substrate. In addition, some amorphous and globular regions were present in minor amounts and they can be attributed to Cu_2_O, typically present on Cu.^48^ Here, we focus on the morphology of the functionalized CVD-G/Cu which appeared to have dot-like structures uniformly formed across the surface of graphene, as shown in Fig. 4a. This granular network, as seen in the zoomed-in image in Fig. 4b, can be attributed to the functionalization of graphene as these dot-like structures are present in both metallic and oxidized regions, compatible with topologies observed for covalent functionalization of HOPG using N-heterocyclic carbenes;^49^ A similar AFM topography was found on HOPG functionalized with 4-nitro phenyl diazonium,^50^ where dendritic growth was observed. Unfortunately, from our data it is not possible to distinguish between dendritic and layer-by-layer growth.

The AFM topography of pristine CVD-G/Cu can be seen in Fig. S15a.† As in the case of the functionalized sample under study, we observed the typical lines found when measuring Cu substrates with AFM as well as island-shaped amorphous structures attributed to Cu_2_O formed underneath the layer of graphene. Because of the rough Cu surface, imaging with AFM is rather challenging; thus Fig. S15a† appears to show areas that cannot be flattened using the software and appear as black regions. As we zoom in over an area of interest, as in Fig. 4b, we observe the same lines of the substrate but no granular network is observed. This may be a further indication that the dot-like structures found in the functionalized sample under study may indeed be due to the functionalization itself. The chlorobenzene molecules bonded through the functionalization process lead to a non-homogeneous (dot-like) film with an average thickness of several nanometers.

### Thermal desorption

**Scheme 2:**
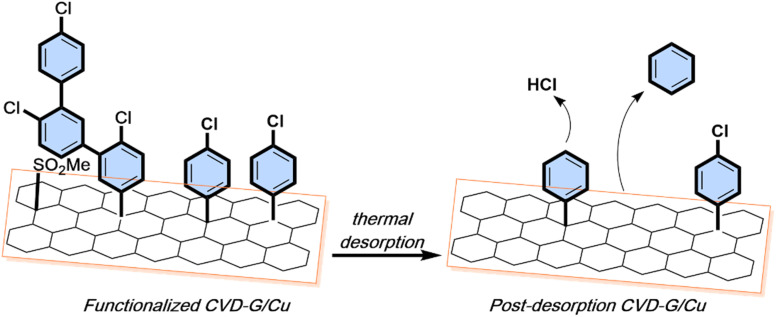
Functionalized CVD-G/Cu surface structure before and after thermal desorption in UHV at 500 °C.

Thermal desorption experiments on post-reaction graphene constitute additional evidence of effective surface functionalization. A linear temperature ramp up to 500 °C allowed measurement of various fragments, including the aryl group (C_6_H_6_^+^/C_6_H_5_^+^) with *m*/*z* 78 and 77 which appeared above 400 °C and HCl with *m*/*z* 38 and 36 (confirmed by the ^35^Cl/^37^Cl isotopic ratio) above 200 °C. In contrast, no significant signal from the chlorobenzene moiety was detected (Fig. SI 21†). The presence of two complementary fragments from chlorobenzene at two different desorption temperatures could indicate the two-step decomposition of the chlorobenzene covalently bonded onto graphene, where first chlorine (in the form of HCl) was desorbed followed by desorption of the benzene ring. Similar results were reported for covalently bonded aryl groups on graphene by chemical activation of arylazo carboxylic *tert*-butyl esters^6^ and electrochemical functionalization *via* diazonium salts (the most common functionalization method) of the phenylsulfanyl group on CVD-G/Cu.^51^ The main *m*/*z* species in these papers were the aryl fragments of benzene (77 or 78), and only minor contributions were observed from the whole molecule used to bond onto graphene. After desorption, XPS spectra showed a significant decrease in the Cl 2p signal together with the transformation of C sp^3^ into C sp^2^, confirmed through a reduction of the Raman D band. In Scheme 2 the structural properties of functionalized CVD-G/Cu are summarized before and after the thermal treatment.

### The role of copper

The surface functionalization process employed in this study exhibited an improved efficiency characterized by rapid kinetics obtained at low concentrations, comparable with that of a previous study reported where visible light was used: 2–5 minutes when benzoyl peroxide is used with a 514.5 nm laser^17^ or only a few seconds with the laser-triggered photolysis of silver trifluoroacetate.^16^ Furthermore, the kinetics were an order of magnitude faster than those reported in prior investigations involving graphene, *i.e.* 2 hours for ultrasonication assisted functionalization,^10^ 21 h for cycloaddition,^52^ 1 hour *via* a perfluorophenyl azide (PFPA)-mediated coupling^53^ or 1/2 hours by using defect-activated sites using arylazocarboxylic *tert*-butyl esters.^6^ However, the kinetics also depend on the substrate; the same functionalization – the covalent immobilization of N-heterocyclic carbenes^49^ – can be achieved in 10 minutes on HOPG or in 24 h in the bulk graphene nanoplatelets. The enhanced efficiency presented in this work is attributed to the role of copper, which influences the reaction dynamics. The interaction between copper and graphene improves the reactivity of the surface, changing its properties according to the surface crystal orientation of copper.^54^

It is well known that a monolayer of graphene absorbs 2.3% of visible light.^55^ This light absorption property stimulates a charge transfer process from the graphene to the arylazo molecule, with formation of a radical and consequent functionalization, in agreement with previous studies on the photochemical reactivity of graphene.^17^ The selection of the wavelength is of great importance in the electron transfer process for both the molecule and graphene, as shorter wavelengths can facilitate electron extraction from the surface. This wavelength-dependent behavior can be rationalized by considering the work function of graphene, which is 4.30 eV for pristine graphene and can be altered by defects, doping, or the substrate used.^56^ The presence of copper beneath the graphene layer has been demonstrated to shift the work function to lower values (3.97–3.81 eV^54^), thereby enhancing the reaction kinetics. Another significant aspect is the presence of oxidized copper regions in pristine CVD-G/Cu. These visible Cu_2_O areas can be observed with an optical microscope (see Fig. SI 8 in the ESI†) and present a darker color than pristine metallic copper. For this reason, it is commonly referred to as a “dark” region (Cu_2_O) in contrast with “bright” regions (metallic Cu). Oxygen can reach copper through the graphene grain boundary and the presence of Cu_2_O has been confirmed by the presence of Raman signals at 149 and 218 cm^−1^ using a 514 nm laser^48^ or 145 and 215 cm^−1^ with a 633 nm laser.^57^ Our Raman spectra (Fig. SI 8†) confirmed the presence of Cu_2_O showing 148 and 218 cm^−1^ signals only in “dark” regions with a 532 nm laser and 142 and 209 cm^−1^ signals with a 473 nm laser. The relative Raman intensity of these Cu_2_O peaks is dramatically affected by the laser wavelength (see Fig. SI 9†). The diffusion of oxygen between the graphene layer and copper forms a large Cu_2_O region, where the graphene is partially detached from the substrate as previously observed by De Luca.^57^ The downward shift of the Raman G and 2D bands to ∼1570 and ∼2650 (Table SI 4†), respectively, observed in these “dark” regions, is compatible with the “freestanding-like graphene”. The copper of pristine CVD-G/Cu is in a metallic “bright” state, but air and humidity exposure lead to the formation of these so-called “dark” (Cu_2_O) regions.

The presence of both Cu states on a macroscopic scale (mm) influences the overall reactivity. Most of the pristine sample was metallic, as assessed by large area (3 × 7 mm^2^) XPS measurements (Cu 2p_3/2_ B. E. at 932.8 eV and Cu LMM at a K. E. of 918.5 eV) previously discussed. Fig. 1 shows the evolution of Raman spectra on the predominantly metallic “bright” regions, but Raman measurements were also made on the “dark” regions of the sample (see Fig. SI 10 and Table SI 4†), where the same evolution of the Raman signal was observed with an *I*_D_/*I*_G_ peak ratio of ∼1.0, indicative of successful covalent bonding. Contrary to “bright” regions, the Raman data for “dark” regions of the control samples also presented a significant increase in the D peak compared to pristine graphene (*I*_D_/*I*_G_ ∼0.2). When the reaction time was extended to 120 minutes, a minor peak (*I*_D_/*I*_G_ ∼1.0) became discernible even in the bright areas. It is crucial to note that accidental irradiation could occur during preparation of the experiments with exposure to ambient natural light. However, this side reaction is not relevant, because it takes place over an order of hours, while the main functionalization reaction is quite fast: after 30 s *I*_D_/*I*_G_ values reach 0.6 over the bright regions (see Fig. SI 11†).

These findings indicate that metallic copper does not actively participate in catalysis without light activation. Conversely, cuprous copper(i) oxide demonstrates the capacity to engage in both reactions, albeit at a slower rate in the absence of light. Thus, we identify Cu(i) as the active species responsible for these reactions. Although aqueous Cu(i) tends to rapidly disproportionate into Cu(ii) and Cu metal if not stabilized using complexing agents, acetonitrile solution of Cu(i) species did not udergo any modification of the chemical state due to specific interaction between the metal ion and acetonitrile^58^ thus allowing rapid electron exchange with any copper(ii) present. In fact, electron transfer in Cu(ii/i) systems is aided by the reorganizational energies involved in changing from a tetrahedral d^9^ Cu(i) four-coordinate system to a six-coordinate d^10^ Cu(ii) one.^59^

Copper chemical states were further investigated using XPS after the reaction to observe the evolution of the Cu oxidation (see Fig. in SI 13†). XPS peaks for Cu 2p, the Auger Cu LMM and O 1s confirmed the presence of partial oxidation of copper from pristine (mainly metallic Cu and Cu(i)) to Cu(ii) after the reaction. Despite this behavior suggesting a Single Electron Transfer (SET) mechanism, it remains unclear whether copper exclusively interacts with the graphene substrate or whether it also directly involves the arylazo sulphone enabling dinitrogen elimination as reported in the literature.^60^ Since the surface exposed to the solution and light is composed of both CVD-G/Cu and pristine copper (localized at the edges of the sample), we are not able to discriminate between these two surfaces. Anyway, the observed oxidation of Cu prompts consideration of the potential release of copper atoms into the solution post-oxidation. Direct observation of copper release was achieved by subjecting a small (10 × 10 mm^2^) silicon substrate with 30 nm of a thermally evaporated copper thin film which was subjected to the same reaction conditions as the CVD-G/Cu samples. The result was the complete dissolution of the thin film in minutes, as shown photographically and from the Raman spectra (see Fig. SI 12†).

Summarizing, copper was a necessary, but not a sufficient element for the covalent bonding reaction. In order to prove this assumption, we performed the same functionalization reaction on a copper-free sample, using the surface of HOPG which is 100% C sp^2^, and compared the results with those of HOPG immersed together with a copper wire. Both experiments were performed in acetonitrile 0.15 mM for several minutes of LED irradiation. The functionalization was extremely effective (*I*_D_/*I*_G_ ∼0.06) and homogeneous over the whole surface only in the presence of the copper wire, while no effective functionalization under copper-free conditions was observed (*I*_D_/*I*_G_ ≪ 0.01). The intensity of the observed *I*_D_/*I*_G_ agreed with that in previous work on HOPG functionalization with diazonium salts^61,62^ and with that in our previous work on HOPG with arylazo sulfones, but at a high concentration (50 mM) and long LED exposure times (24 h).^12^

**Scheme 3:**
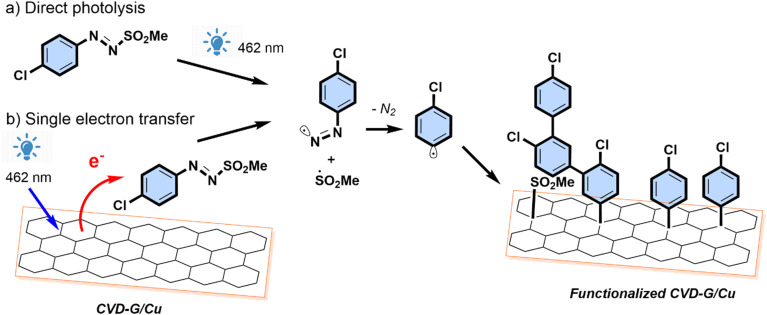
Photoreaction mechanisms of arylazo sulfones on CVD graphene on Cu.

The proposed mechanism is illustrated in Scheme 3, wherein incident light simultaneously initiates the activation of the graphene surface and the cleavage of arylazo sulfones. This dual process results in the elimination of molecular nitrogen and the generation of aryl and 
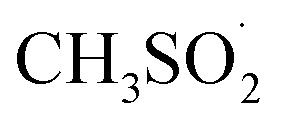
 radicals which subsequently bond to the graphene surface, leading to surface functionalization. An alternative pathway for radical generation involves indirect activation attributed to charge transfer from copper. A similar mechanism is given by the single-electron transfer living radical polymerization (SET-LRP), where a radical anion initiator [Pn/P–X]˙^−^ interacts with Cu(0) and other copper electron donors to generate Pn˙ and X^−^ as Cu_2_O is formed. Cu(0) undergoes conversion *via* single-electron transfer to Cu(i), which then undergoes disproportionation into Cu(0) and Cu(ii), establishing a cyclic process that is deactivated by the generation of Cu(ii).^63^ The reaction is strongly affected by the solvent and it has a reduced rate in non-disproportionating solvents such as acetonitrile.^64^

The control of covalent functionalization using visible light offers a novel method for the fabrication of patterned arrays and could overcome the current approach consisting of the deposition of resist materials with successive cycles of lithography and electrochemistry,^65^ or be an alternative to using oxygen-sensitive photosensitizers such as iodonium salts.^29^ Although direct patterning has been successfully employed for quantum dots,^66^ produced through covalent reactions with a chosen ligand, no successful attempts have been reported for visible-light, specific photo-induced covalent bonding of generic organic molecules on surfaces such as silicon, gold or graphene. One of the versatile aspects of such wavelength-specific photo-induced reactions on surfaces lies in the possibility of generating diverse reactive species even from the same starting molecule based on the wavelength of the light sources used.

## Conclusions

We have presented a novel rapid photo-functionalization method for CVD graphene on copper using arylazo sulfones. The functionalization was proven by UV-Vis, Raman and XPS analyses showing the formation of covalent bonding on graphene. Furthermore, thermal desorption was carried out and the mass spectra of fragmentation during the heating process confirmed the covalent functionalization of the graphene surface. The efficiency of the method was primarily attributed to copper's role in enhancing the process, with the metal present as metallic copper (bright region) and cuprous oxide (dark region). Copper was able to activate the reaction for radical attack on the graphene surface, while insights into the molecular photo-dissociation provided help in understanding the overall mechanism.

The resemblance between this mechanistic pathway and the observed experimental findings presented in this work suggests the possibility for a photocatalytic enhancement of SET-LRP, using visible light and azosulfones. The approach could be reiterated by using different building blocks ultimately allowing fine tuning of graphene surfaces by molecular engineering with arylazo sulfones for micropatterning applications.

## Data availability

The data supporting this article have been included as part of the ESI.† Atomic composition of samples obtained by X-ray photoelectron spectroscopy and all the main parameters of Raman spectra analysis are reported in the ESI.†

## Author contributions

A. M. performed the functionalization and the desorption experiments and acquired Raman, UV-Vis and XPS spectra. A. M. and A. K. analyzed the Raman, XPS and Auger signals. V. B. acquired and analyzed the AFM topography. A. K. conceptualized and supervised the research. A. M., A. K., D. J., M. B., M. M. and V. P. discussed the reaction mechanism and conditions. The manuscript was written with contributions from all authors. All authors have given approval to the final version of the manuscript.

## Conflicts of interest

There are no conflicts to declare.

## Supplementary Material

NA-OLF-D4NA00359D-s001
