# Supplementary material for: Covalent functionalization by using blue light activated radicals: on the reaction mechanisms of arylazo sulfone binding on graphene
Source: Nanoscale Adv. 2024 Aug 1;6(20):5080–91. doi: 10.1039/d4na00359d (PMC11333948; doi:10.1039/d4na00359d)
Supplement: NA-006-D4NA00359D-s001 [file NA-006-D4NA00359D-s001.pdf]

Supplementary Information (SI) for Nanoscale Advances.
This journal is © The Royal Society of Chemistry 2024

Supporting Information

Covalent functionalization by using blue light activated radicals: on the reaction mechanisms of Arylazo Sulfones binding on Graphene.

Alessandro Mameli [1,2], Alessandro Kovtun[2], Derek Jones[2], Vasiliki Benekou[3], Vincenzo Palermo[2], Marco Bandini [1], Manuela Melucci[2]

Table of Contents

General Method....2
Raman characterization of molecules....3
UV-Vis and stability 6
UV-vis light activation of Arylazo sulfones by LED....9
Raman characterization of CVDG/Cu....14
XPS....19
AFM....20
Functionalization of HOPG....21
Thermal desorption of functionalized graphene....24
References....30

General Method

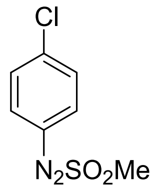

4-chloro-phenylazo methyl sulfone

Pale yellow solid; yield = 92%. Spectral data match those reported in the literature [1].

Commercially available chemicals were purchased from Sigma Aldrich and Fluorochem and used without any further purification.

High resolution XPS spectra of C 1s were analysed by CasaXPS (Casa software, Ltd), the curve fitting was carried out using asymmetric pseudo-voigt for the aromatic C-C sp^2 component with fixed asymmetry of 0.14 [2], a FWHM of 1.2 eV and centred at 284.4 eV. All the other chemical state were fitted by symmetric pseudo-Voigt with 1.4 eV FWHM and the subtraction of Shirley background was done prior the fit. Since the relatively high complexity of heteroatoms bonded to carbon in functionalized samples (Nitrogen, Oxygen, Sulphur and Chlorine) the synthetic components used for C 1s fit were grouped and only 4 were used: i) C−C sp^3, defects and aryl ring at 285.1 eV (Chemical shift of +0.7 eV); ii) C-O and C-Cl at 286.2 eV; iii) C=O in carbonyl or carboxyl at 288.6 eV. C-N contribution was not clearly associable to a component in C 1s fitting, given the relatively low relative abundance, thus it was not possible to understand if the contribution was from single or double bonded C-N or other combinations. Only the functionalized sample that was treated at 500°C present an extra peak at 291 eV, that can be associated to a shake-up transition.

More detail on consistency of the fitting procedure is reported in our previous work on C 1s fit [2].

N 1s signal in functionalized sample was fitted by two components at 400.4 and 398.6 eV (not reported), that are compatible with C-N=N-C unit (399.4 eV from [3]), but also to most of the other C-N chemical species.

Thermal desorption was performed by using HAL 3F-RC, a triple filter quadrupole with Thorium-Iridium filament, with Faraday Cup detector for AMU from 1 to 50 and Electron Multiplier for range 51-200 AMU. Electron energy was set to 70 eV. All mass spectra of functionalized graphene were compared to the mass spectra of pristine graphene at the same temperature, the considered mass peak – and thus mass fragments due to functionalization - were identified by subtracting the spectra of functionalized and pristine graphene.

Raman characterization of molecules

The analysis of the Raman spectra of 4-chlorophenylazo methyl sulfone was done by using literature and computational software KnowitAll™. It should be underlined that a precise assignment of all peaks could be reached only with more analysis and the present constitutes only a preliminary assignment of the vibrational motions.

Figure SI 1 shows the molecule Raman spectra of 4-chlorophenylazo methyl sulfone between 300 and 1800 cm-1. Though N≡N stretching vibration occurs around 2294 cm^{-1} [4][5], no discernible signals were detected beyond 1800 cm^{-1}, thus this spectral range has been excluded from our analysis.

In the Raman spectrum, distinctive peaks provide insights into the molecular vibrations of SO_2. The peak at 516 cm^{-1}, attributed to wagging, and the peak at 1131 cm^{-1} are identified as SO_2 molecular vibrations

Several peaks at 1406 cm^{-1}, 1467 cm^{-1}, and 1484 cm^{-1} are assigned to "Combination bending + stretching aromatic ring". These indicate a combination of bending and stretching motions within an aromatic ring structure.

Exploring further, specific vibrational modes associated with aromatic rings are discerned. The peak at 627 cm^{-1} corresponds to in-plane deformation involving C=C-C bonds. In agreement with Socrates [5], the bands shift to a higher wavenumber for para-substituted aromatic compounds compared to mono-substituted compounds.

At 886 cm^{-1}, the "ring breathing" vibrational mode suggests cyclic structures undergoing expansion and contraction.

Moving to 1088 cm^{-1}, the assignment is doubtful. In literature, there is a complex mode identified as "CH in-plane bending for p-substituted and mono-substituted benzenes coupled with C-N stretching" for diazonium salts. This phenomenon is explained by electron-charge density redistribution within the structures caused by the presence of N_2^+. For the azosulfones, the net charge is neutral but there could be a partial change acting in a similar way and creating this peak.

At 1193 cm^{-1}, the vibrational mode is characterized as "m; o CH in-plane bending," signifying in-plane bending motions of CH bonds in meta (m) and ortho (o) positions. Finally, at 1585 cm^{-1}, the "C=C stretching" mode represents the stretching motion of a C=C bond. According to Wojciechowski [6], this band is essentially related to ortho-meta C-C stretching modes.

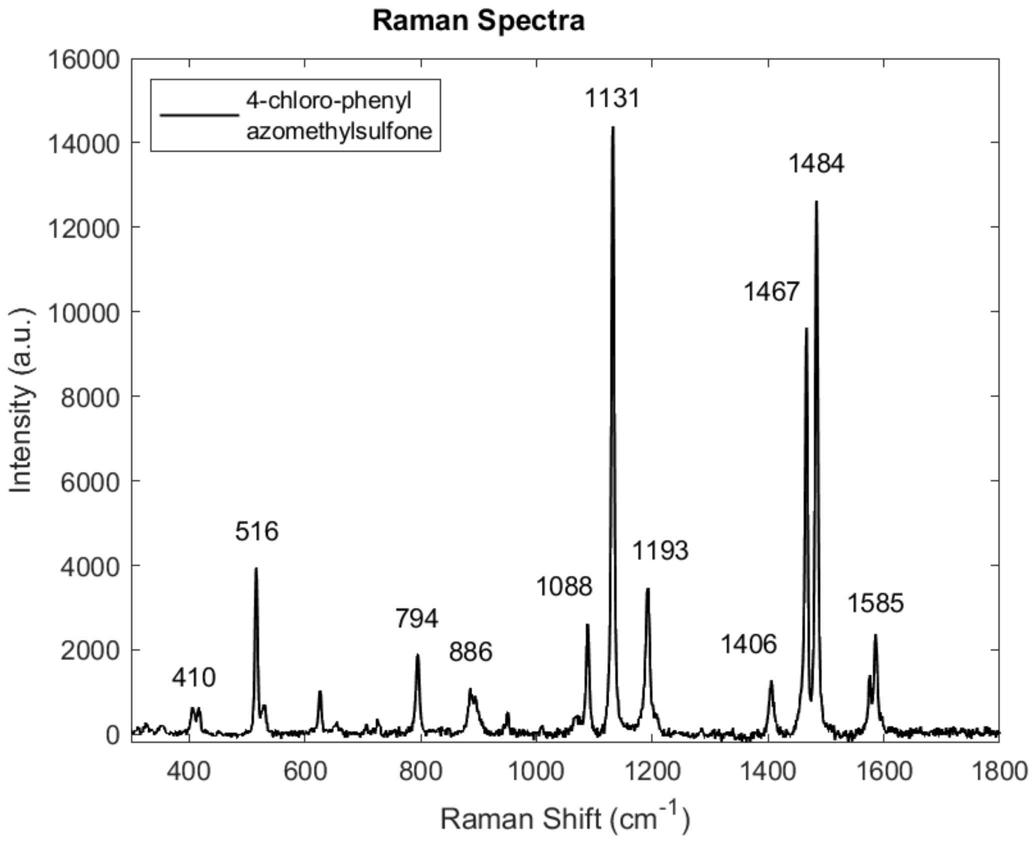

Figure SI 1. Raman spectra of 4-chloro-phenylazo methyl sulfone with assignment of peaks. Spectrum acquired with 633 nm laser from dry powder deposited on glass.

Table SI 1. Assignment table of 4-chloro-phenyl azomethylsulfone.

Peak (nm)	Assignment
410	Not assigned
516	SO_2 wagging (KnowitAll)
627	Ring deformation (C=C-C in plane deformation) [4]
794	Not assigned
886	Ring breathing [4]
1088	CH in plane bending for p-substituted and mono- substituted benzenes [7][5] coupled with C-N stretching
1131	SO_2 (KnowitAll)
1193	m; o CH in plane bending [4]
1406	Combination bending + stretching aromatic ring (KnowitAll)
1467	Combination bending + stretching aromatic ring (KnowitAll)
1484	Combination bending + stretching aromatic ring (KnowitAll)
1585	Benzene ring C=C stretching [4]

UV-Vis and stability

UV-vis spectra of 4-chloro-phenyl azomethylsulfone in ACN were recorded over a concentration range of 0.05 to 0.25 mM as depicted in Figure SI 2a-b. Within the concentration range of 0.2 to 0.1 mM, distinct absorbance peaks were observed at 305 nm and 425 nm, corresponding to π-π* and n-π* transitions, respectively. All samples were consistently prepared at a concentration of 0.15 mM, that is in accordance with previously reported values in literature of 0.1mM for grafting of gold [8].

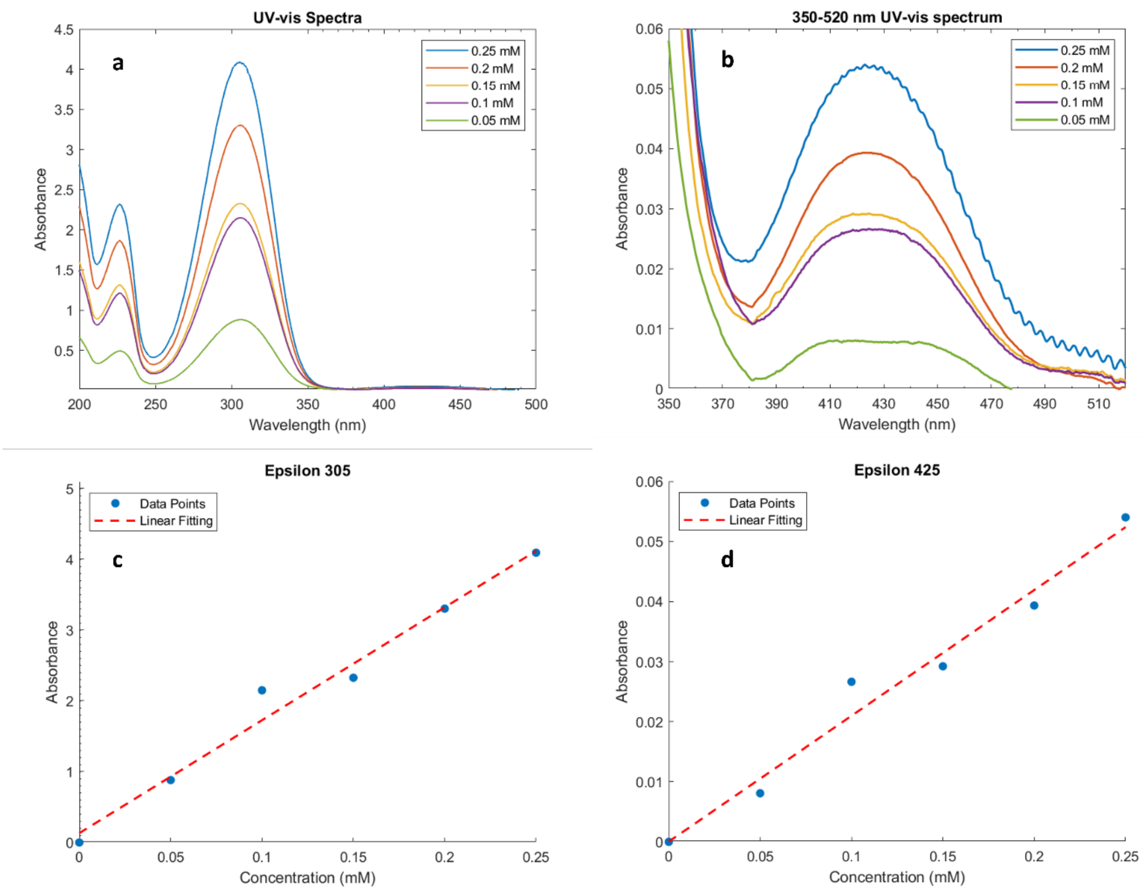

Figure SI 2. (a) UV-Vis spectra for 0.05-0.25 concentration range of 4-chlorophenylazo methyl sulfone. (b) 425 nm band attributed to n-π* transition. (c) Molar absorptivity fit for 305 nm band (d) Molar absorptivity fit for 425 nm band.

UV-Vis spectra were utilized to determine the molar absorptivity (ε) of 4-chlorophenylazo methyl sulfone for both the 305 nm (Figure SI 2c) and 425 nm (Figure SI 2d) peaks. ε_{305} and ε_{425} values were determined by plotting the maximum absorbance against concentration, where found to be 16000 and 210 M^{-1} cm^{-1}, respectively. The obtained coefficients align with the order of magnitude observed in literature data for arylazo sulfones [9].

Photodegradation experiments on of 4-chlorophenylazo methyl sulfone were conducted by exposing a 0.15 mM sample of 4-chlorophenylazo methyl sulfone to laboratory illumination conditions (Neon lamps and no direct Sun exposure) over a two-month duration. UV measurements were acquired at various time points: 1 day, 3 days, 15 days, 30 days, and 60 days. Figure SI 3 displays the UV-Vis spectra illustrating the absorption characteristics of 4-chlorophenylazo methyl sulfone over time.

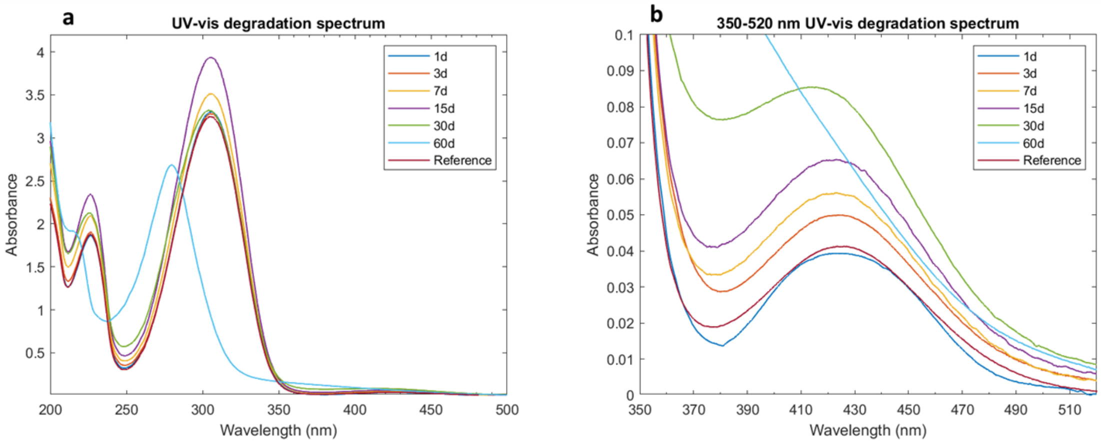

Figure SI 3. Photodegradation of 4-chlorophenylazo methyl sulfone in the 200-600 nm spectral interval and (b) detail in 330-550 nm visible light region.

Table SI 2. Peak center shift after 60 days degradation

Reference (nm)	60 days (nm)
226.5	215.5
305.0	282.5
425.0	415.5

The solution exhibited stability during the initial 15 days. Subsequently, from 30 days onwards, a gradual change in absorbance was observed, likely attributed to the formation of radicals and the introduction of additional light-absorbing species. The most noticeable shift occurred after 60 days, predominantly within the 280 to 310 nm region. In Table SI 2 these spectral shifts are presented.

Given the overall stability of the solution, experiments were conducted using solutions that were less than two weeks old, or, whenever possible, with freshly prepared solutions.

UV-vis light activation of Arylazo sulfones by LED

Reaction kinetics of 4-chlorophenylazo methyl sulfone and CVD-G/Cu were studied in a range between 5 and 45 minutes (Figure SI 4a). We observed from UV-Vis spectra that absorbance in the region 200-270 nm (Figure SI 4b) grows and the peak center moves from 220 to 210 nm. In the other two regions, the change is less clear. In the region 240-340 nm (Figure SI 4c), ascribed to a π-π* transition, the initial band before reaction seems to be formed by two different bands with inverse proportional growth, visible only after the reaction. The two bands peaked approximately at 280-320 nm and 340 nm have an opposite evolution in time: while the absorbance of band at 280-320 nm decrease quite rapidly until the collapses after 45 minutes, the band at 340 nm slowly rise during the whole considered time. The last region 370-530 nm (Figure SI 4d), ascribed to a n-π*, presents an overall increase in intensity, due to formation of radicals and it is probably composed by multiple components, not clearly identificable. In Azobenzenes the exitation of π-π* UV band brings to a isomerization from cis to trans [10], with an increase of π-π* band and a decrease of n-π* band, while in our molecule the effects seems to be almost the opposite: illumination and consequent increase of the n-π* band, while π-π* band decreases; that is associated in literature [11] to a partial isomerization (from trans to cis in 4-chlorophenylazo methyl sulfone) before the release of the sulfone radical. Radical formation and isomerization could both be present, but for a quantitative detection of radical an EPR spectroscopy might

be helpful.

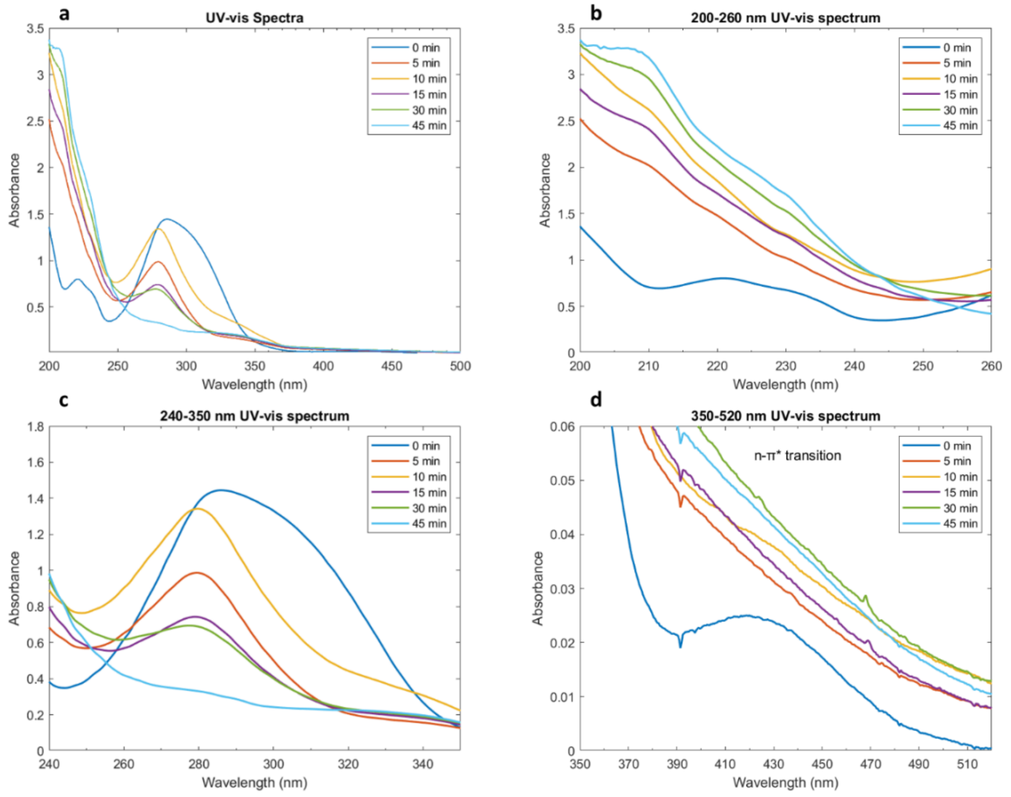

Figure SI 4. Time evolution of UV-Vis spectra for CVD-G/Cu irradiated for 0-5-15-30-45 minutes in the range (a) 200-600, (b) 200-260, (c) 240-340, (d) 330-540 nm.

General procedure for the light promoted functionalization of CVD-G/Cu

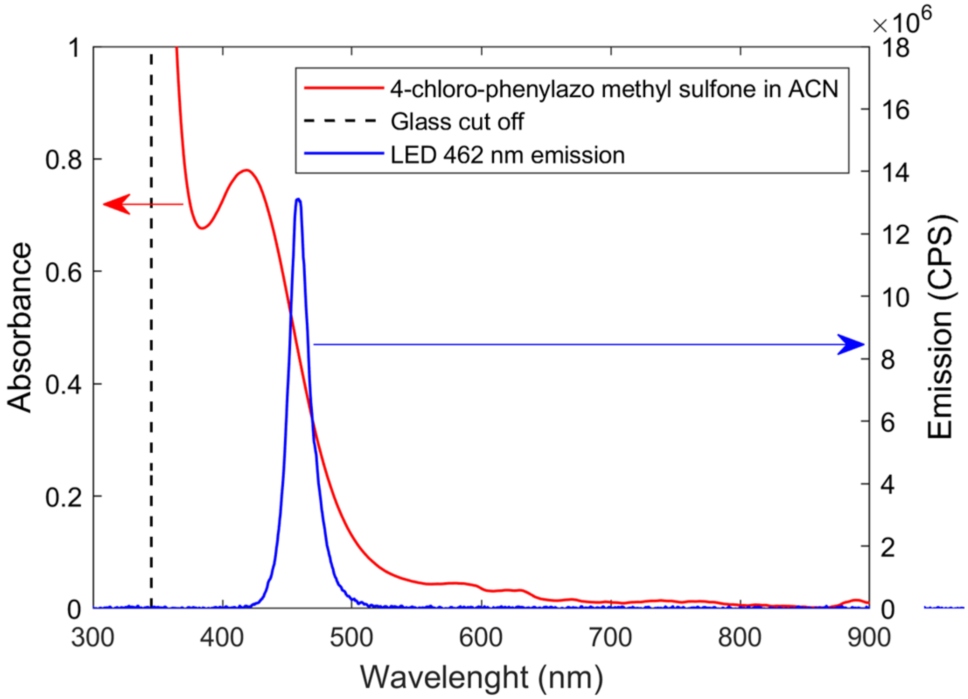

Figure SI 5. Comparison between absorbance of 4-chlorophenylazo methyl sulfone (red curve – left y axis) and LED emission (blue curve – right axis).

The photofunctionalization reaction described in Scheme 1 was performed using a solution of 4-chlorophenylazo methyl sulfone in pure ACN, utilizing an excitation light source with a wavelength of 462 nm and different surfaces as substrates.

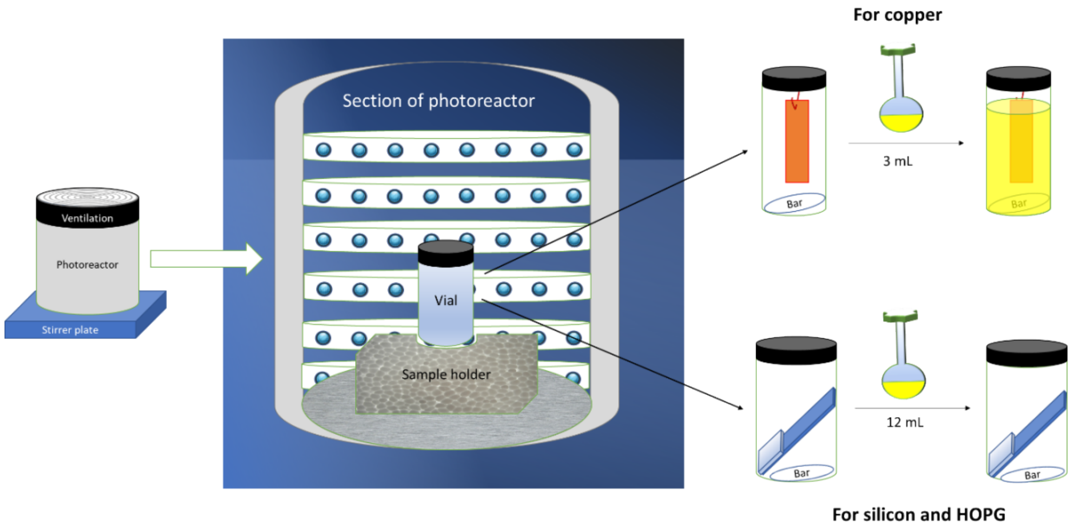

Figure SI 6. Scheme of the reaction set-up with a detailed zoom inside the photoreactor. On the right, detail on vials. Copper reaction use a wire to attach the surface. Other substrates rely on a diagonal plate.

As depicted in Figure SI 6, the experimental set-up for photofunctionalization consisted of a stirrer plate and a cylindrical photoreactor. Within the photoreactor, a sample holder was set on a polystyrene support to maintain vial stability. Surrounding the interior of the photoreactor, single commercial LED strips were affixed, comprising a total of 300 LEDs with an electrical power of 23W (12V – 1.9A). This configuration resulted in an approximate current of 20mA for each LED, and more than 90% of electric power was converted into light, the remaining 10% was dissipated by joule effect on resistances present on LED strip. The emission spectrum of the LEDs was characterized by a UV-Vis spectrometer, revealing a single emission peak at 462 nm (Figure SI 7e), consistent with the typical emission of commercial Gallium Nitride LEDs. The 23W LED strip had the secondary effect of elevating the temperature within the reactor. To maintain the temperature at approximately 30 °C, a PC cooling vent was installed atop the reactor. The irradiation distance between the LED source and the vial was maintained at approximately 10 cm.

In Figure SI 6, the vials used for the reaction are illustrated on the right part. For the experiments, a 50 mL of 4-chlorophenylazo methyl sulfone stock solution was prepared (Figure SI 7a). The functionalization of copper substrates (pristine copper and CVD-G/Cu) was executed using 4 mL vials (Figure SI 7b), into which 3 mL of the 4-chlorophenylazo methyl sulfone solution were transferred. A copper wire was suspended from the vial cap, ensuring that the stirrer bar did not come into contact with the substrate.

For HOPG, a volume of 12 mL was transferred to a 16 mL vial. Inside this vial, a rectangular piece of silicon wafer was diagonally positioned to prevent direct contact between the sample and the stirrer bar. Subsequently, the vial was placed inside the photoreactor, and the reaction proceeded under magnetic stirring and LED illumination for a fixed time. Following the reaction, the substrate was removed from the solution, rinsed with ACN, and promptly dried using a flow of nitrogen gas.

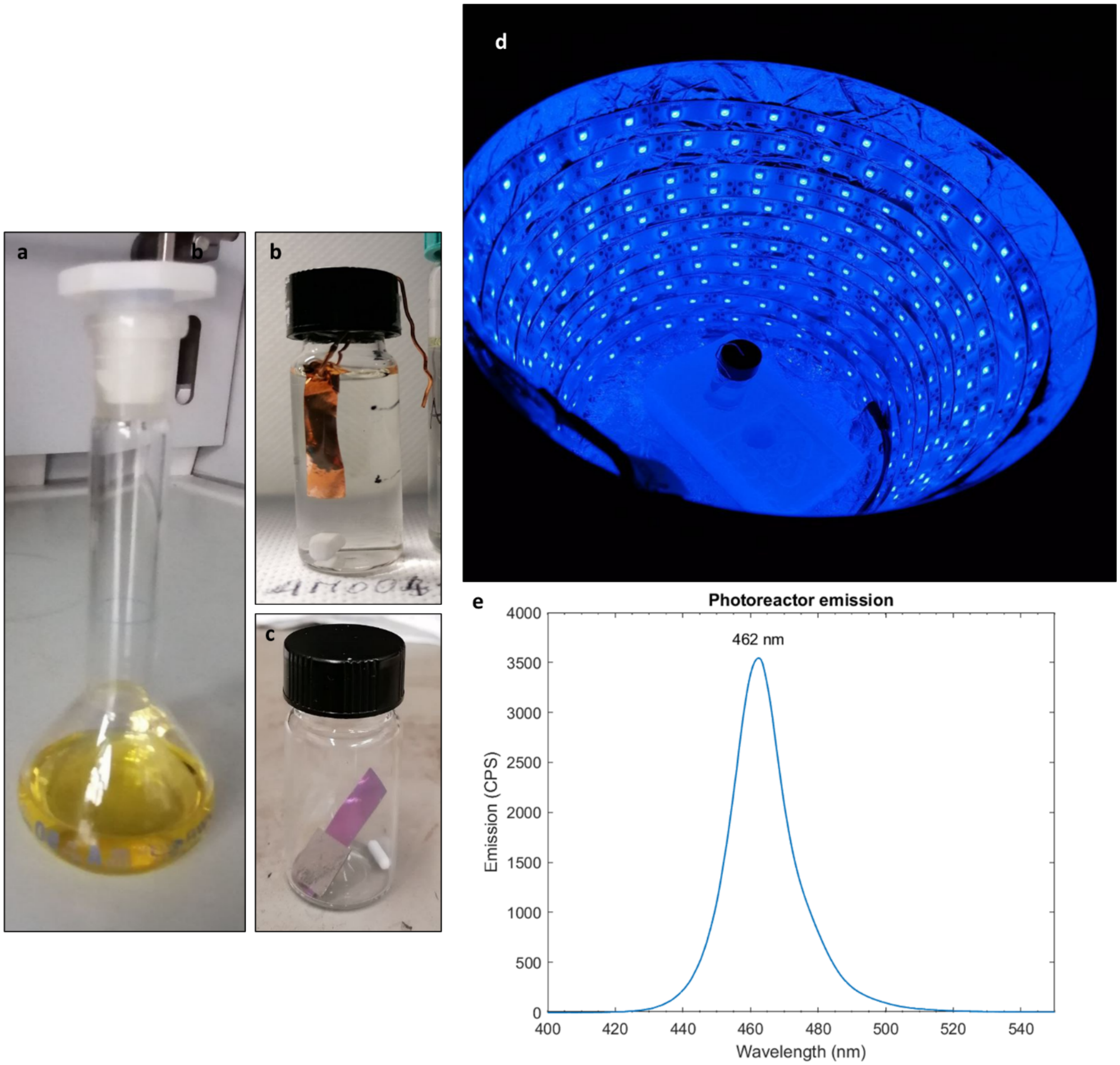

Figure SI 7. (a) Stock solution of 4-chlorophenylazo methyl sulfone in ACN (0.15mM in 50 mL). (b) Reaction support for copper sample, volume is 4 mL. (c) Reaction support for HOPG, silicon and gold sample, volume is 16 mL. (d) Photoreactor during the reaction. (e) Emission spectrum of the used LED source.

Raman characterization of CVDG/Cu

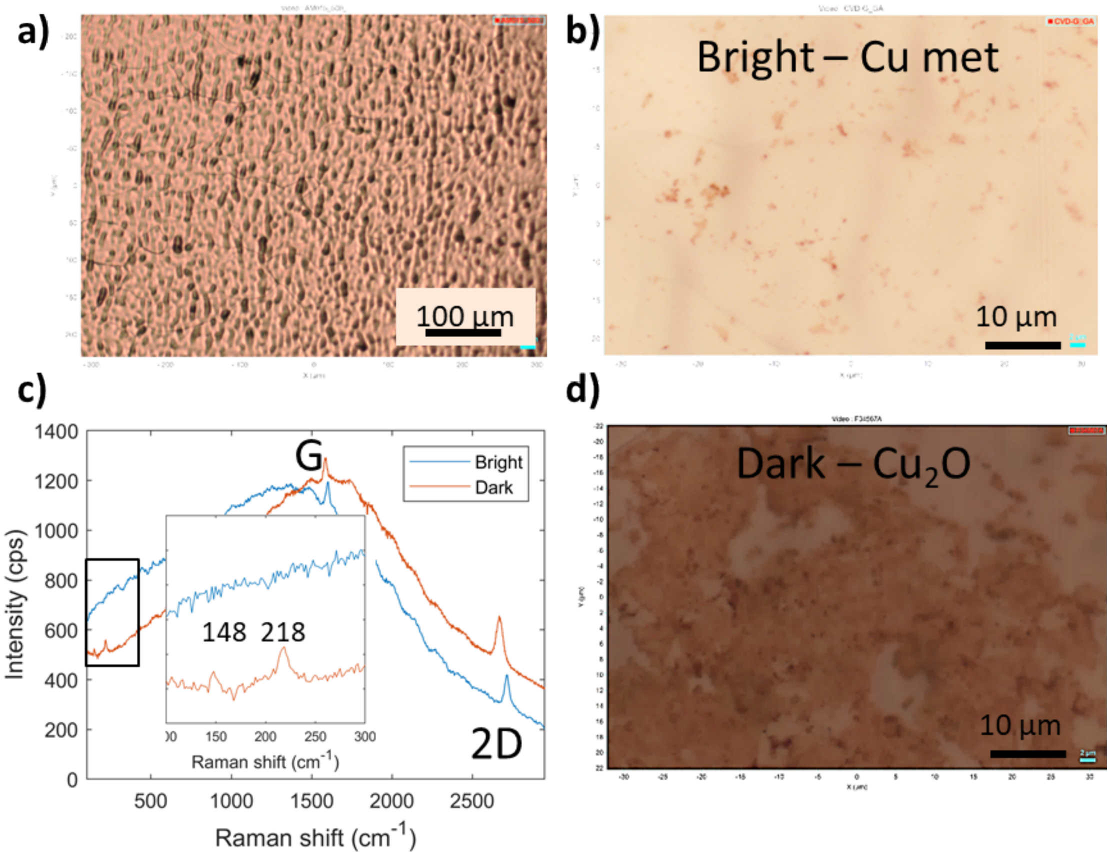

Figure SI 8. Pristine CVD-G/Cu. (a) Optical Miscopy image at low magnification (10x) and high magnification (100x) of "Bright" region. (c) Representative Raman Spectra of "Bright" regions (Metallic Cu) and a "Dark" region (Cu_2O), inset: 100-300 cm^{-1} interval with Cu_2O fingerprint signals. (d) High magnification image of a "Dark" region.

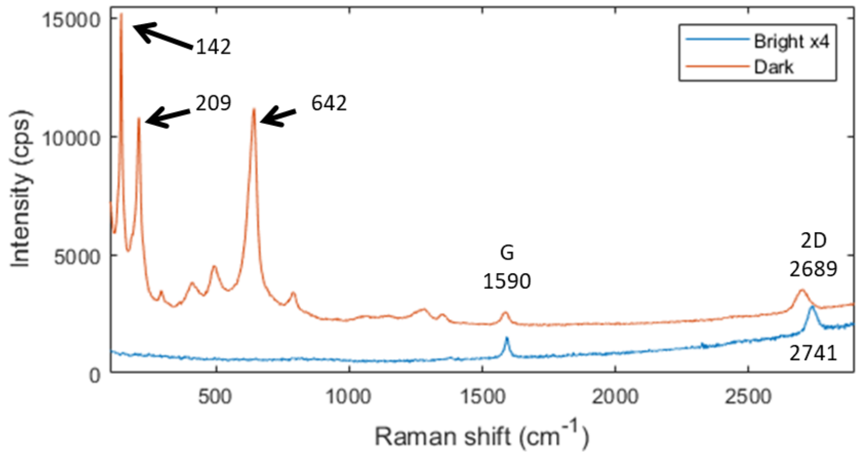

Figure SI 9. Representative Raman Spectra of “Bright” regions (Metallic Cu) and a “Dark” region (Cu_2O), acquired with 473 nm Laser (100x, 0.5mW power).

Table SI 3. Spectral parameters obtained from fit of Pristine, control and functionalized CVD-G/Cu in dark region. 10 spectra for each sample type were acquired in different spots along the sample.

Parameter	Pristine	Control	Functionalized
D (cm^{-1})	1346±8	1338±6	1323±8
G (cm^{-1})	1572±6	1579±4	1582±6
2D (cm^{-1})	2648±10	2669±10	2632±10
I_D/I_G	0.12±0.02	0.20±0.15	1.0±0.3
FWHM 2D (cm^{-1})	50±5	42±4	68±4
A_{2D} / A_G	5.2±0.9	5.2±0.6	~0.1

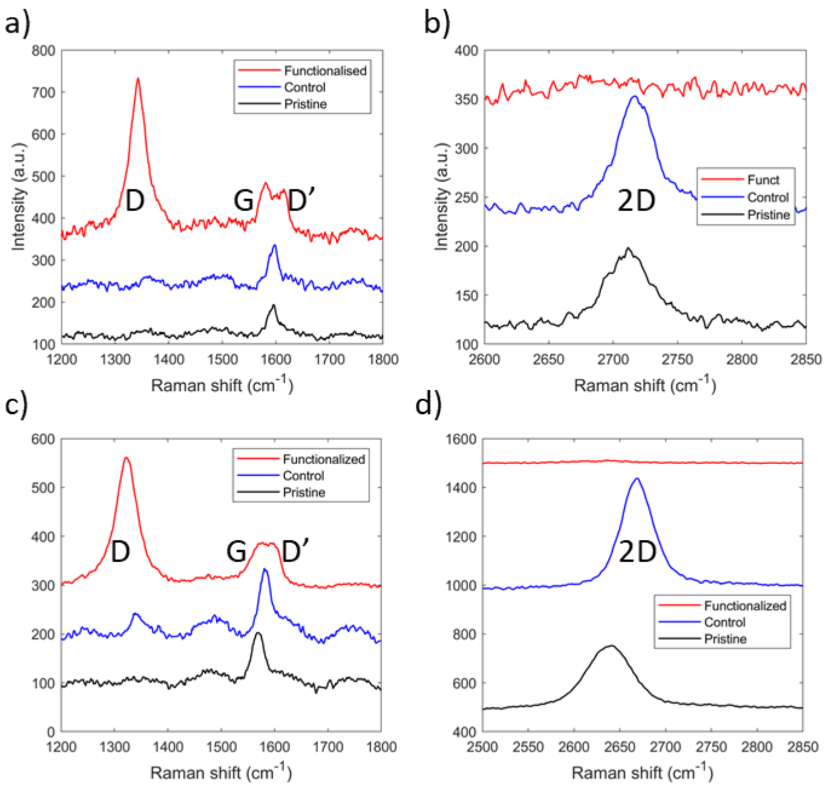

Figure SI 10. CVD-G/Cu functionalized by 4-chlorophenylazo methyl sulfone (ACN, 0.15 mM, 30°C, 7 min 23W LED). Representative Raman spectra after background subtraction of (a,b) Bright regions (Metallic Cu) and (c,d) Dark Regions (Cu_2O).

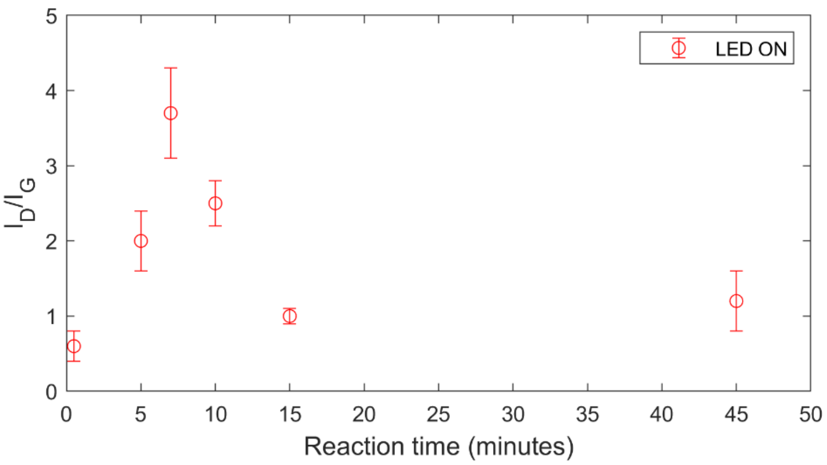

Figure SI 11. ID/IG ratio evolution of CVD-G/Cu functionalized by 4-chlorophenylazo methyl sulfone (ACN, 0.15 mM, 30°C). Only "bright" metallic region was considered.

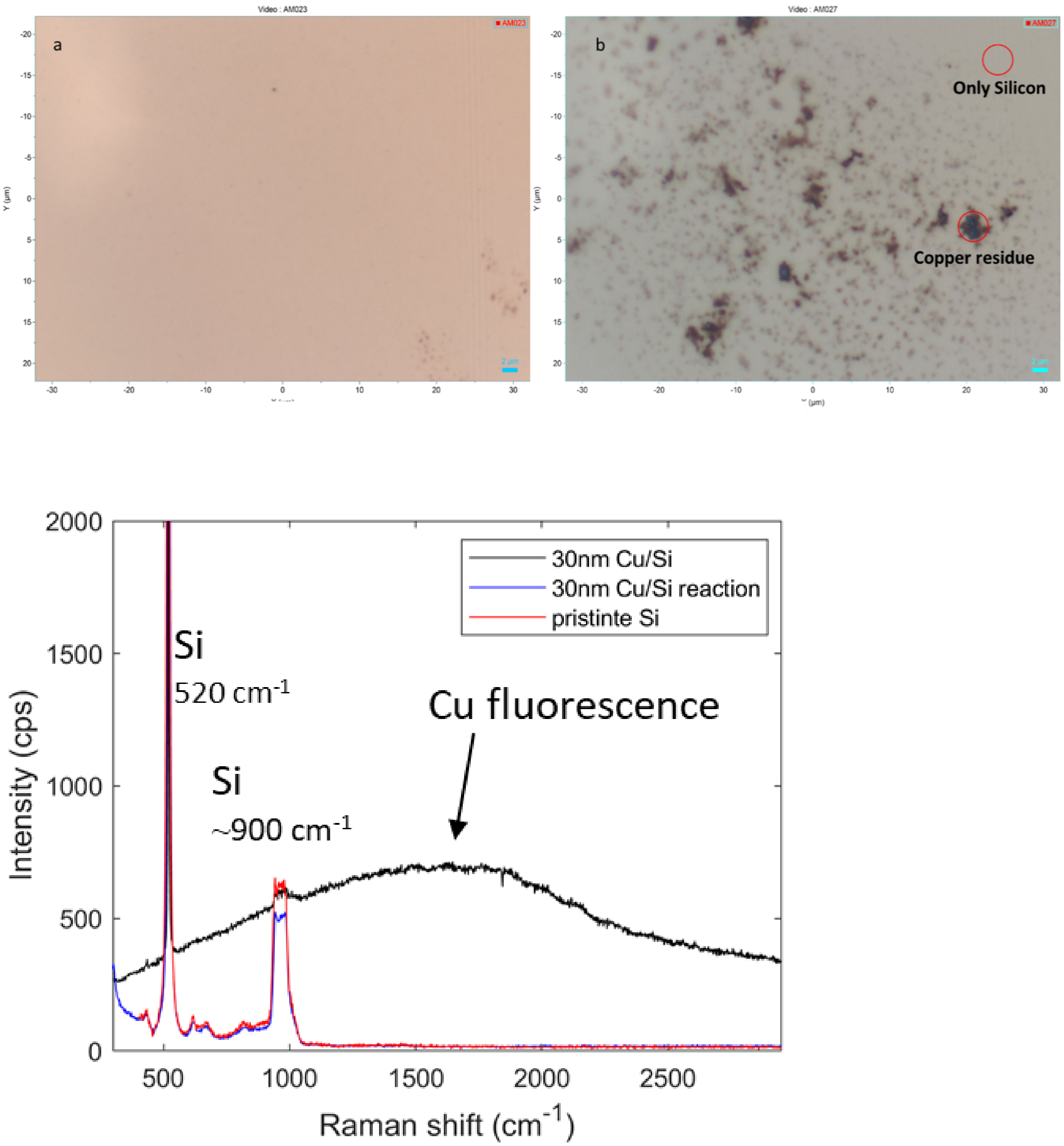

Figure SI 12.(a) Optical image at 100x of pristine Cu on a Si substrate. (b) Optical image at 100x of the substrate post 7 minute reaction with 4-chlorophenylazo methyl sulfone with the two different zone circled in red. (c) Raman spectra of a pristine Silicon wafer (red line), of 30nm of copper deposited on Silicon wafer (30nm Cu/Si) and the same 30nm Cu/Si after 7 minute of reaction (ACN, 0.15 mM, 30°C, 7 min 23W LED).

XPS

The XPS spectra presented in Figure SI 13 provide insight into the behavior of copper and oxygen in pristine, control, and 7-minute functionalized samples. In pristine sample, copper exists predominantly in the metallic state, evidenced by the binding energy of 932.8 eV for Cu $2p_{3/2}$ and the kinetic energy of Auger Cu LMM signal at 918.5 eV, with a corresponding Auger modified parameter of 1851.3 eV. In control sample the presence of Cu(I) as Cu_2O is confirmed by the binding energy of 932.3 eV in Cu $2p_{3/2}$, matching that of the metal, and the kinetic energy of Auger Cu LMM signal at 916.8 eV [12], with a corresponding Auger modified parameter of 1849.1 eV. The effective presence of Cu_2O was further supported by the O 1s signal, which peaked at 530.6 eV [13] as shown in Figure SI 13c.

Despite both pristine and control samples exhibiting the same profile for Cu $2p_{3/2}$, corresponding to a mixture of Cu(I) and Cu(0), they display a distinct profile for Cu LMM, which points to an oxidation shift from mostly Cu(0) to Cu(I). This discrepancy may be attributed to the oxidizing capability of 4-chlorophenylazo methyl sulfone.

Upon comparing the control and functionalized samples, the rise of novel peak in the Cu $2p_{3/2}$ signal at 934-935 eV was observed only in the irradiated – thus functionalized - sample, indicative of Cu(II) presence. This secondary oxidation is attributed to light exposure. Furthermore, the emergence of shake-up features in the Cu 2p signal (945-942 eV), the modification of the Cu LMM signal (a broad peak compatible with Cu(I) and Cu(II) plus a sharp peak correspondent to metallic Cu), and an increase in the O 1s signal (532 eV compatible with Cu-OH at 531.8 eV in ref. [13]) collectively support the overall presence of copper in the Cu(II) state, mainly in hydroxide form of $Cu(OH)_2$ on surface, mixed with some residual metallic and oxides regions.

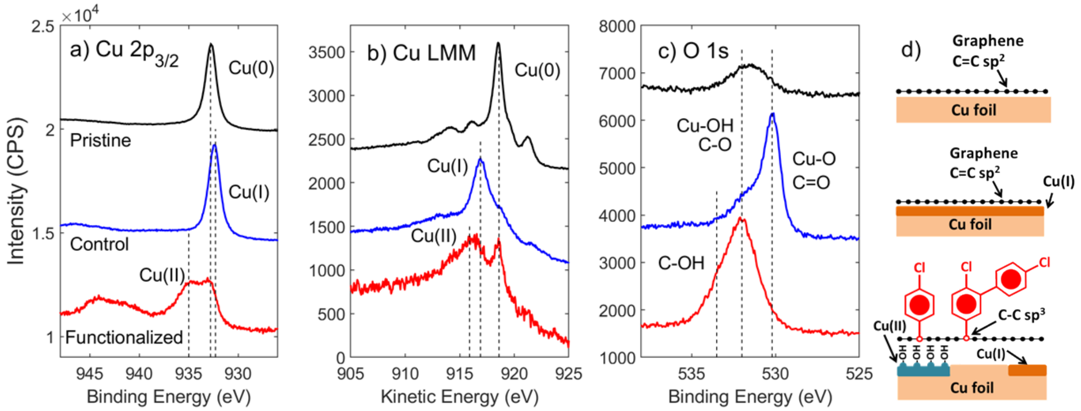

Figure SI 13. XPS spectra of pristine, control and functionalized CVD-G/Cu. (a) XPS Cu $2p_{3/2}$ signal. (b) Auger Cu LMM signal. (c) XPS O 1s signal. (d) Scheme of possible chemical state at graphene-copper interface. The functionalized sample was irradiated at 23W LED power.

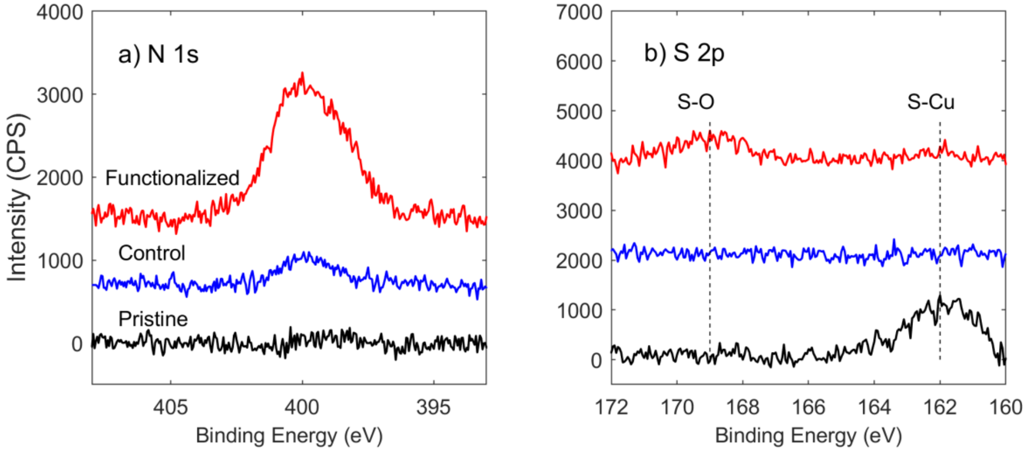

Figure SI 14. XPS N 1s (a) and S 2p (b) spectra of pristine, control and functionalized CVD-G/Cu.

AFM

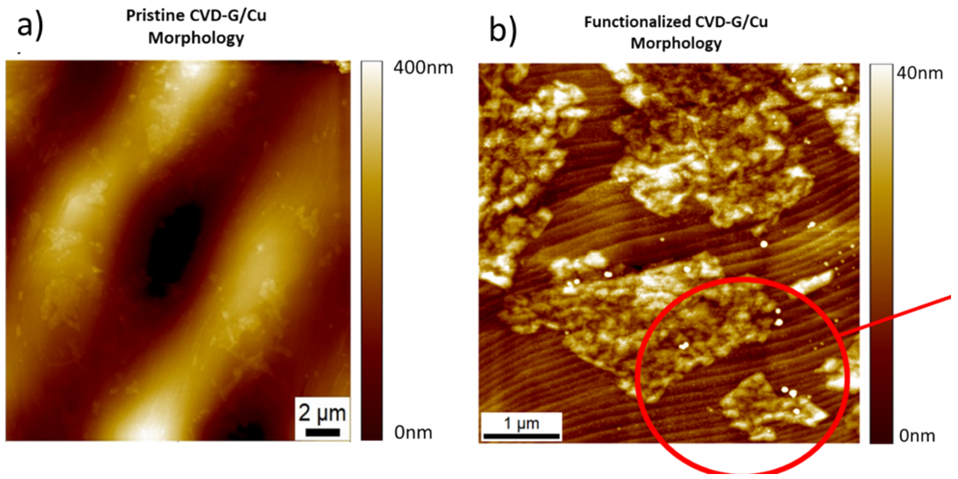

Figure SI 15. AFM topography of pristine CVD-G/Cu (a) and after the functionalization (b). The globular agglomerates were associated to the oxidised region of copper.

Functionalization of HOPG

The functionalization of HOPG was controlled by using Raman spectroscopy, the pristine HOPG sample presen a G peak at 1580±1 cm^{-1}, while 2D in the 2712-2713 cm^{-1} range. D signal was not detected, but was expected in the range between 1346 and 1350 cm^{-1}. The values are in good agreement with established literature for HOPG measured by 532 nm laser.

Raman spectroscopy shows that the covalent photografting performed in condition similar (0.15 mM for 15 minutes) to the one employed for CVD-G/Cu does not occur with HOPG, unless a Cu wire is introduced during the reaction (Figure SI 16a). The low concentration (0.15 mM) and relatively short irradiation time (15 minutes) are not enough, contrary to the successful grafting on HOPG in high concentration (50 mM) and long time (24h) reported in our previous work [14].

Another important parameter that can catalyze the reaction is the temperature. Performing the reaction for 24h at 50 mM and keeping the temperature at 30°C produces no visible functionalization, while raising the temperature to 40° C yields a D peak (Figure SI 16b).

For the sample at 40° C, the surface was inspected in several areas and three different spots are shown, finding that D peak is present only in areas in which structural defects are present (Figure SI 17).

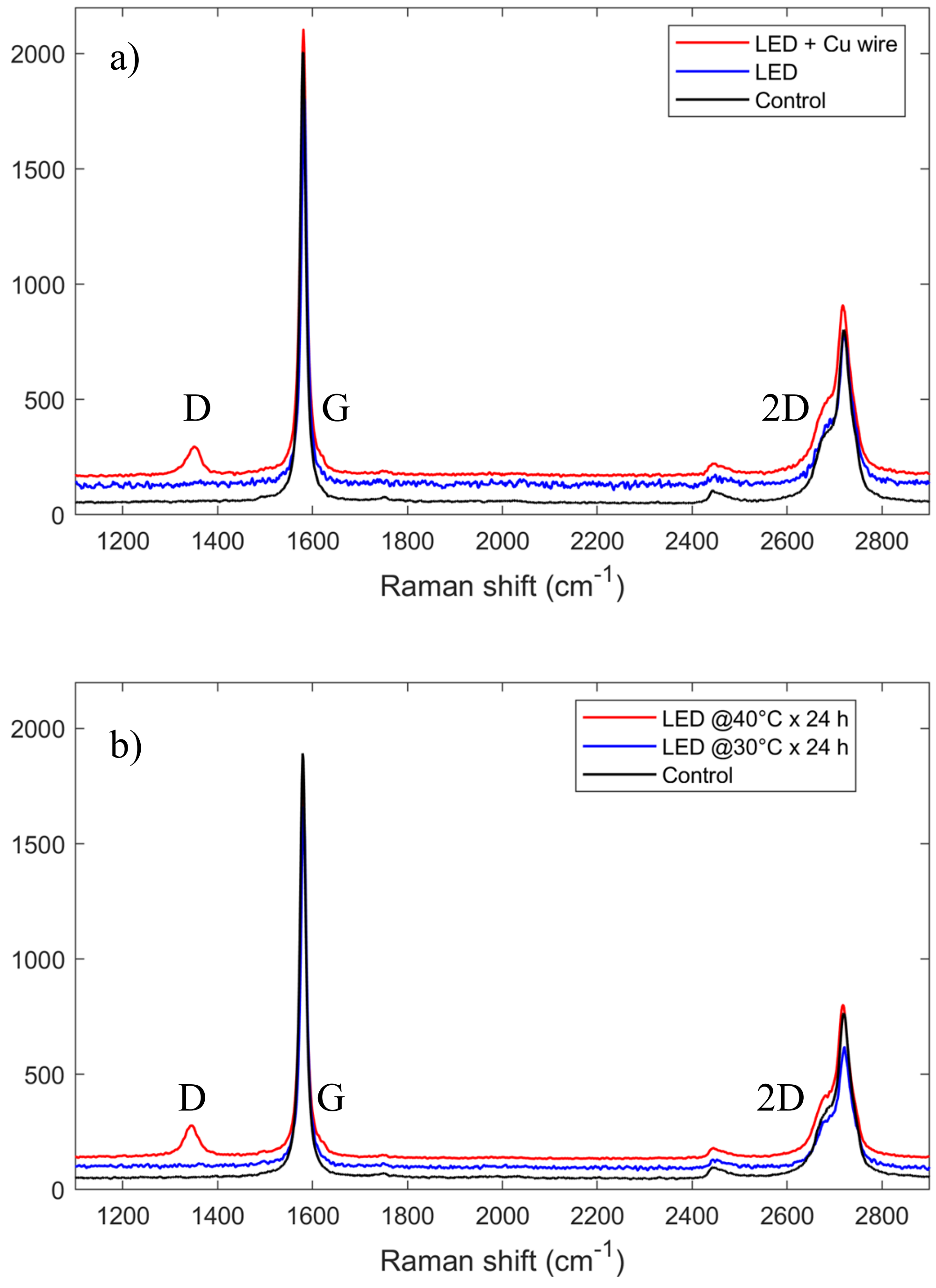

Figure SI 16. (a) HOPG functionalized by 4-chlorophenylazo methyl sulfone (ACN, 0.15 mM, 30°C, 15 minutes). Representative Raman spectra of reaction with and without the presence of Copper wire in the reaction solution. (b) HOPG functionalized by 4-chlorophenylazo methyl sulfone (ACN, 50 mM, 24 h). Representative Raman spectra of reaction at 30°C and 40°C.

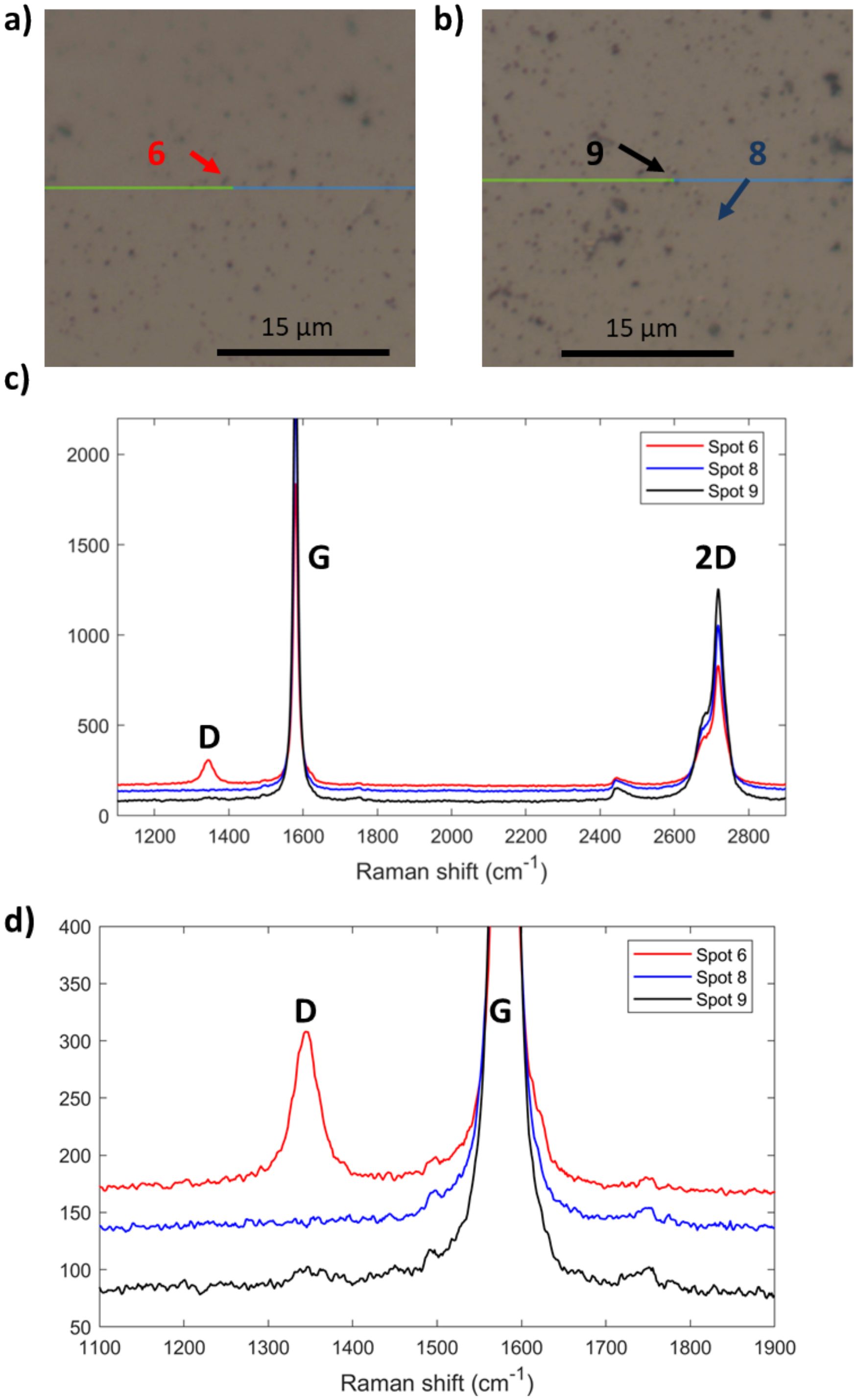

Figure SI 17. HOPG functionalized by 4-chlorophenylazo methyl sulfone (ACN, 50 mM, 40°C, 24h). (a) and (b) optical microscope image at 100X of functionalized HOPG, (c) and (d) Raman spectra acquired in 3 different areas: surface defects in spot 6 (ID/IG = 0.08) and 9 (ID/IG = 0.00)

Thermal desorption of functionalized graphene

Thermal desorption experiments were conducted on samples functionalized for 7 minutes on CVD-G/Cu, comparing pristine (referred to Pristine + 500° C) and functionalized samples (referred to as Functionalized+500°C). A thermal ramp at 5°C per minute from room temperature to 500°C was employed, closely controlled by PID. Prior to the experiment, the sample was placed in a vacuum chamber at $3x10^{-8}$ mBar for one day. XPS spectra were acquired before and after the desorption process.

XPS spectra of the functionalized CVD-G/Cu post-desorption were analyzed, and the atomic composition are reported in Table SI 4 and are compared with the control desorption experiment (Pristine + 500° C) performed on pristine CVD-G/Cu (Figure SI 22). XPS Cu 2p spectra in Figure SI 18a-b demonstrate an overall reduction of Cu from Cu(II) to Cu(0) after thermal desorption, so the copper has returned to the metallic state similar to the pristine condition. XPS analysis also reveals a decrease of chlorine and nitrogen presence on the surface (Figure SI 18e), suggesting partial de-functionalization.

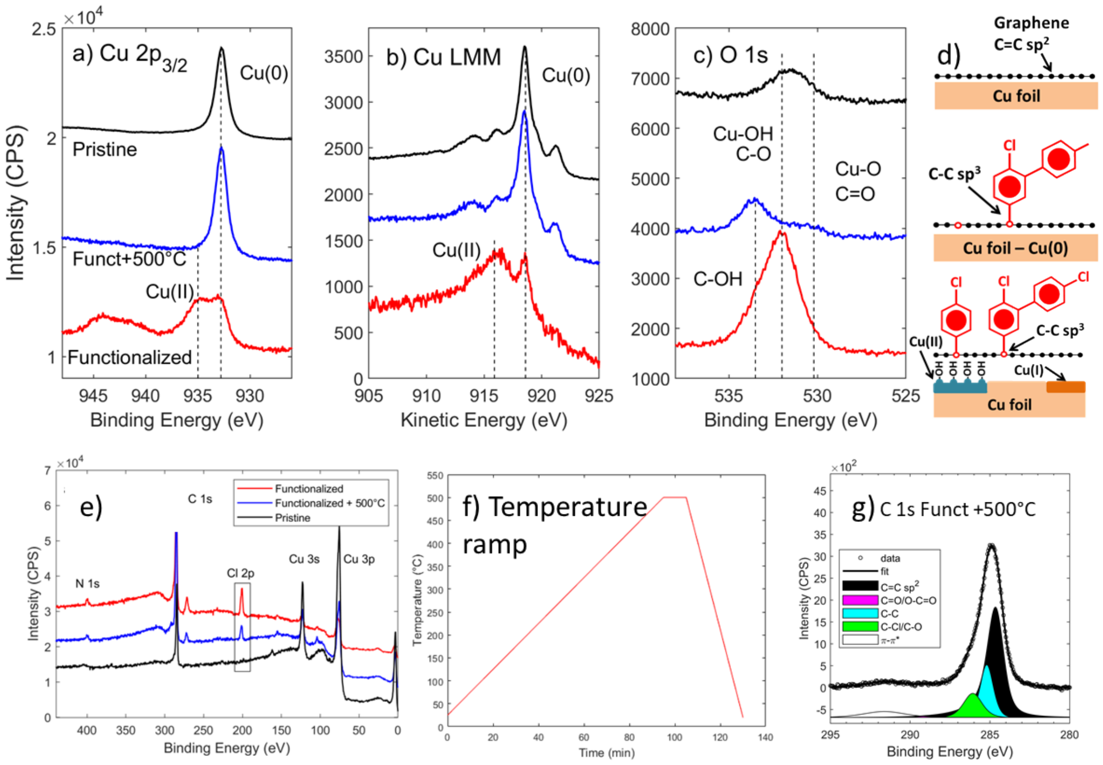

Figure SI 18. XPS of CVD-G/Cu functionalized by 4-chlorophenylazo methyl sulfone (ACN, 0.15 mM, 7min) and heated in UHV to 500°C.

Carbon KLL signal and the corresponding D parameter value for pristine, functionalized, and post-desorption samples is shown in Figure SI 19. Notably, the D parameter increases in both pristine and functionalized samples. This indicates that desorption at 500°C effectively has reduced the covalently grafted chlorobenzene on the surface and it mostly restored the pristine state of sp^2 aromatic carbon.

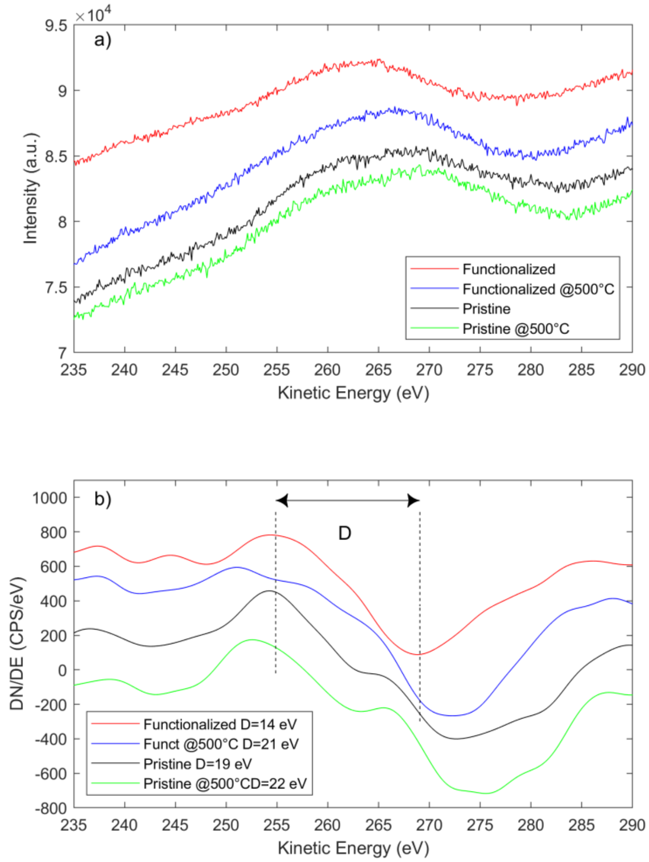

Figure SI 19. C KLL signal of CVD-G/Cu functionalized by 4-chlorophenylazo methyl sulfone (ACN, 50 mM, 30°C, 24h) and then heated in UHV to 500°C. The same thermal treatment was performed on pristine CVD-G sample.

Table SI 4. XPS thermal desorption data comparison between pristine+500 and functionalized+500

XPS signal	Binding Energy (eV)	chemical state	Pristine After 500°C (at.%)	Functionalised After 500°C (at.%)
C 1s	284.4	C=C/C-C sp2	33.3	52.4
	285.1	C-C sp3	-	15.9
	286.3	C-Cl	-	10.3
	288.7	C=O / O-C=O	-	0.8
O 1s	532.0	O-C / Cu-OH	0.5	3.1
	530.6	O-Cu / O=C	1.0	0.8
Cu 2p 3/2	932.8	Cu(0)	63.9	11.4
	932.3	Cu(I)	-	-
	935.0	Cu(II)	-	-
Cl 2p 3/2	200.5	Cl-C	-	3.2
N 1s	400	N-C	-	1.6
S 2p 3/2	161.6	S-Cu	1.4	0.4
	168.6	S-O	-	-

Figure SI 20a displays bright and dark region of CVD-G/Cu, along with a new area formed by interconnected points, possibly nucleation sites generated predominantly within the dark region through chlorobenzene decomposition by heating. Raman spectra for the three zones exhibit the same profile and shape, thus we presented only one desorbed sample in Figure SI 20b.

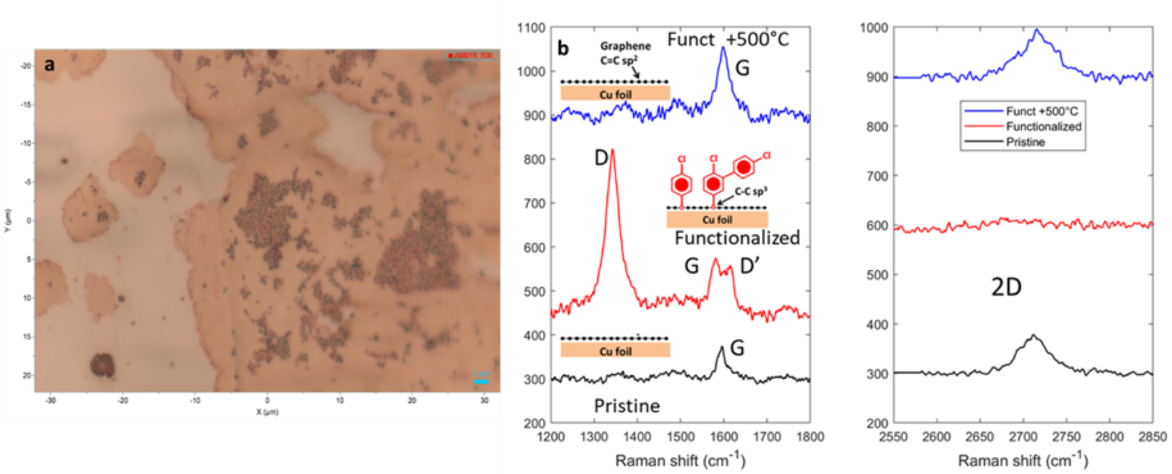

Figure SI 20. Raman of CVD-G/Cu functionalized by 4-chlorophenylazo methyl sulfone (ACN, 0.15 mM, 30°C, 7min) and heated in UHV to 500°C.

The desorbed sample spectrum reveals a significant decrease of the D peak (<0.2), to the increase of A_{2D}/A_G ratio above 2 in dark region (both dark and dots), albeit with a larger FWHM (<50 cm^{-1}) and close to 1 in bright regions. Apart from some minor differences the Raman spectra is compatible with monolayer/bilayer graphene, but it's behind this work the investigation of these graphene-based material.

Moreover, Raman spectroscopy results imply that the applied temperature was insufficient to achieve complete surface re-annealing. Prior research conducted by Kidambi et al. [15] has demonstrated that complete re-annealing in CVD pristine surfaces is marked by an increase in the intensity of the D peak. In contrast, our results indicate only a marginal increase when starting from a pristine sample and no increase when commencing with a functionalized sample.

Finally, a mass spectrometry fragmentation analysis was conducted, and the results are depicted in Figure SI 21. The mass distribution of the fragments is consistent with the expected ratios of $Cl^{35}/Cl^{37}$for Cl in HCl (the correspondent ratio of m/z=36 over m/z=38 is

plotted in Figure SI 21f). However, a poor correlation is observed with the chlorobenzene fragment (Figure SI 21a-b). The presence of additional fragments at increasing temperatures is reported in Figure SI 21g-i.

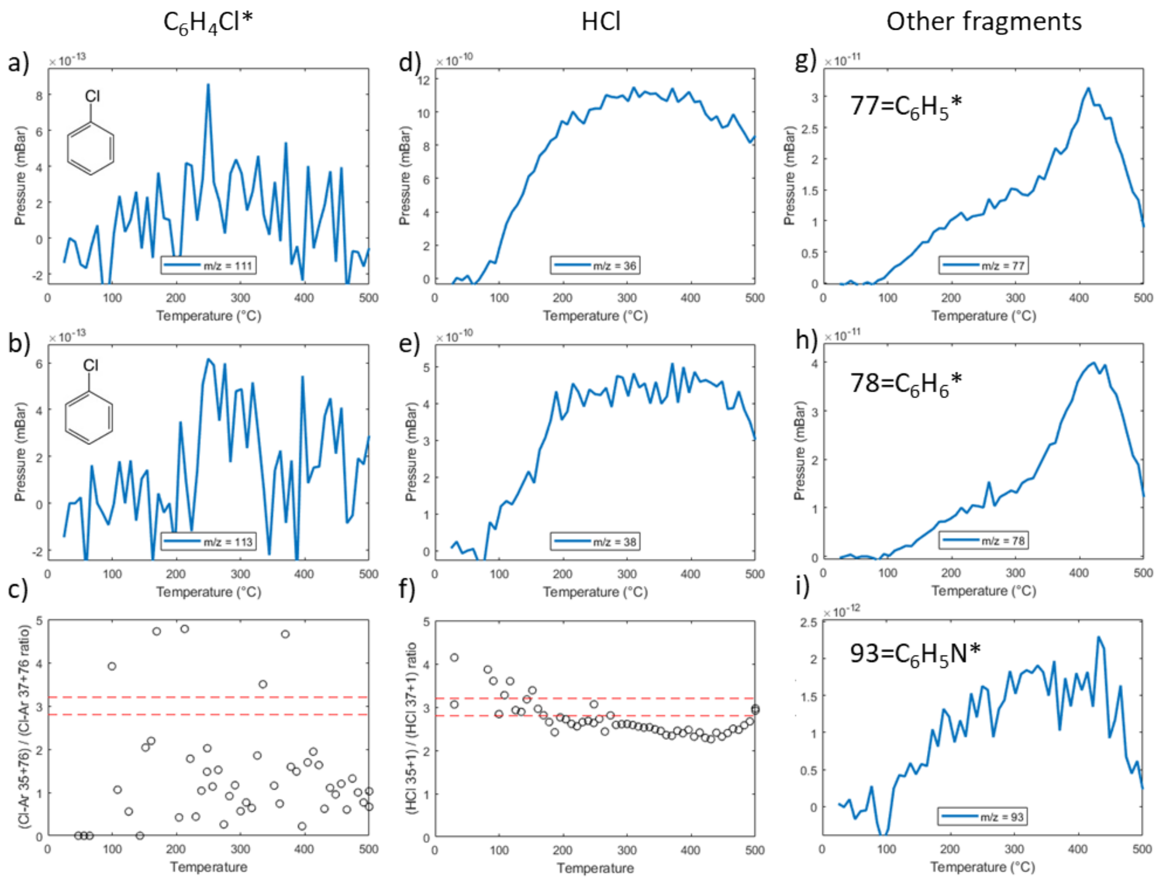

Figure SI 21. CVD-G/Cu functionalized by 4-chlorophenylazo methyl sulfone (ACN, 0.15 mM, 30°C, 7min) and heated in UHV to 500°C. (a,b) Quadrupole signal for fragment with m/z=111 and 113, correspondent to chlorobenzene and (c) ratio between m/z = 111 and 113, dotted line: expected value for Cl isotopes (~3). (d) $^{1}H^{35}Cl$ signal (m/z=36), (d) $^{1}H^{37}Cl$ signal (m/z=38) and (d) ratio between m/z=36 and m/z=38. (g-h) fragment from aromatic ring and (i) from C_6H_5N*.

The control desorption experiments were performed on pristine CVDG/Cu, where only minor modifications were observed, mainly due to oxygen desorption, which was originally present between graphene and copper, as previously reported by Kidambi [15].

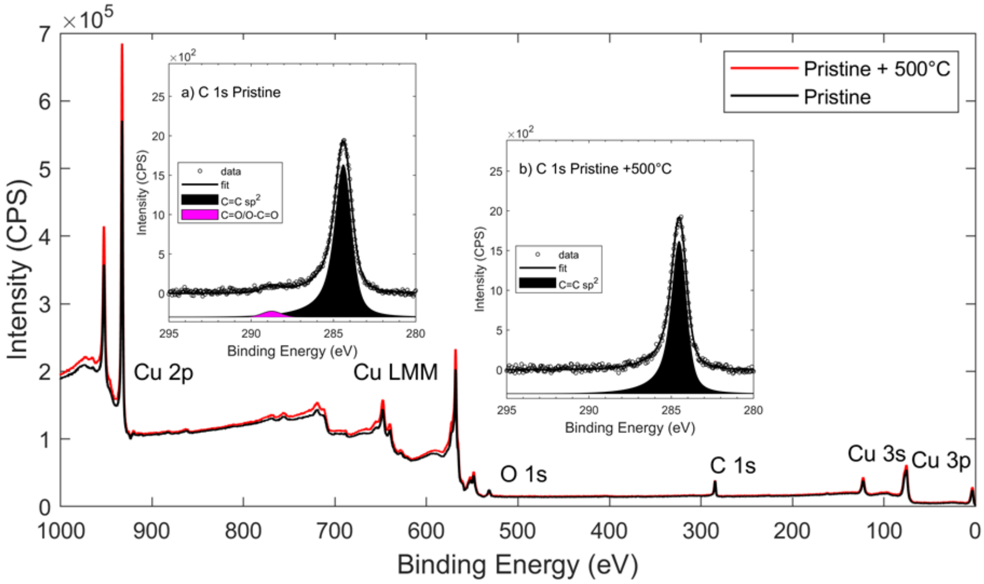

Figure SI 22. XPS of pristine CVD-G/Cu before and after the thermal treatment in UHV at 500°C. Insets show the C 1s signal before (a) and after (b) the treatment.

References

[1] C. Lian *et al.*, "Visible-Light-Driven Synthesis of Arylstannanes from Arylazo Sulfones," *Org. Lett.*, vol. 21, no. 13, pp. 5187–5191, 2019, doi: 10.1021/acs.orglett.9b01788.

[2] A. Kovtun, D. Jones, S. Dell'Elce, E. Treossi, A. Liscio, and V. Palermo, "Accurate chemical analysis of oxygenated graphene-based materials using X-ray photoelectron spectroscopy," *Carbon N. Y.*, vol. 143, pp. 268–275, 2019, doi: 10.1016/j.carbon.2018.11.012.

[3] T. Yoshida and S. Sawada, " The X-Ray Photoelectron Spectroscopy of o -Hydroxy Aromatic Azo Metal Complexes ," *Bull. Chem. Soc. Jpn.*, vol. 48, no. 1, pp. 345–346, 1975, doi: 10.1246/bcsj.48.345.

[4] H. M. Badawi, W. Förner, and S. A. Ali, "A comparative study of the infrared and

Raman spectra of aniline and o-, m-, p-phenylenediamine isomers," *Spectrochim. Acta - Part A Mol. Biomol. Spectrosc.*, vol. 112, pp. 388–396, 2013, doi: 10.1016/j.saa.2013.04.075.

[5] D. A. Long, " Infrared and Raman characteristic group frequencies. Tables and charts George Socrates John Wiley and Sons, Ltd, Chichester, Third Edition, 2001. Price £135 ," *J. Raman Spectrosc.*, vol. 35, no. 10, pp. 905–905, 2004, doi: 10.1002/jrs.1238.

[6] P. M. Wojciechowski, W. Zierkiewicz, D. Michalska, and P. Hobza, "Electronic structures, vibrational spectra, and revised assignment of aniline and its radical cation: Theoretical study," *J. Chem. Phys.*, vol. 118, no. 24, pp. 10900–10911, 2003, doi: 10.1063/1.1574788.

[7] L. Laurentius *et al.*, "Diazonium-derived aryl films on gold nanoparticles: Evidence for a carbon-gold covalent bond," *ACS Nano*, vol. 5, no. 5, pp. 4219–4227, 2011, doi: 10.1021/nn201110r.

[8] J. Médard, P. Decorse, C. Mangeney, J. Pinson, M. Fagnoni, and S. Protti, "Simultaneous Photografting of Two Organic Groups on a Gold Surface by using Arylazo Sulfones as Single Precursors," *Langmuir*, vol. 36, no. 11, pp. 2786–2793, 2020, doi: 10.1021/acs.langmuir.9b03878.

[9] S. Crespi, S. Protti, and M. Fagnoni, "Wavelength Selective Generation of Aryl Radicals and Aryl Cations for Metal-Free Photoarylations," *J. Org. Chem.*, vol. 81, no. 20, pp. 9612–9619, 2016, doi: 10.1021/acs.joc.6b01619.

[10] L̕. Vetráková, V. Ladányi, J. Al Anshori, P. Dvořák, J. Wirz, and D. Heger, "The absorption spectrum of: Cis -azobenzene," *Photochem. Photobiol. Sci.*, vol. 16, no. 12, pp. 1749–1756, 2017, doi: 10.1039/c7pp00314e.

[11] D. Franzke, B. Voit, O. Nuyken, and A. Wokaun, "Pulsed ultraviolet laser photolysis of substituted phenyl azosulfonates wavelength dependent effects," *Mol. Phys.*, vol. 77, no. 2, pp. 397–409, 1992, doi: 10.1080/00268979200102511.

[12] J. F. Moulder, W. F. Stickle, P. E. Sobol, and K. D. Bomben, "Handbook of XPS.pdf." p. 255, 1995.

[13] E. Bojestig, Y. Cao, and L. Nyborg, "Surface chemical analysis of copper powder used in additive manufacturing," *Surf. Interface Anal.*, vol. 52, no. 12, pp. 1104–1110, 2020, doi: 10.1002/sia.6833.

[14] L. Lombardi *et al.*, "Visible-Light Assisted Covalent Surface Functionalization of Reduced Graphene Oxide Nanosheets with Arylazo Sulfones," *Chem. - A Eur. J.*, vol. 28, no. 26, 2022, e202200333. doi: 10.1002/chem.202200333.

[15] P. R. Kidambi *et al.*, "Observing graphene grow: Catalyst-graphene interactions during scalable graphene growth on polycrystalline copper," *Nano Lett.*, vol. 13, no. 10, pp. 4769–4778, 2013, doi: 10.1021/nl4023572.
